# Exploring barriers to and facilitators of malaria prevention practices: a photovoice study with rural communities at risk to *Plasmodium knowlesi* malaria in Sabah, Malaysia

**DOI:** 10.1186/s12889-023-16173-x

**Published:** 2023-07-10

**Authors:** Nurul Athirah Naserrudin, Pauline Yong Pau Lin, April Monroe, Richard Culleton, Sara Elizabeth Baumann, Shigeharu Sato, Bipin Adhikari, Kimberly M. Fornace, Rozita Hod, Mohammad Saffree Jeffree, Kamruddin Ahmed, Mohd Rohaizat Hassan

**Affiliations:** 1 Department of Public Health Medicine, Faculty of Medicine, Universiti Kebangsaan Malaysia, 56000 Kuala Lumpur, Malaysia; 2 Faculty of Medicine and Health Sciences, Borneo Medical and Health Research Centre, Universiti Malaysia, 88400 Sabah, Kota Kinabalu Malaysia; 3 Sabah State Health Department, Ministry of Health, 88590 Kota Kinabalu, Malaysia; 4 Faculty of Social Sciences and Humanities, Universiti Malaysia Sabah, 88400 Kota Kinabalu, Malaysia; 5 Johns Hopkins Center for Communication Programs, Baltimore, MD USA; 6 Division of Molecular Parasitology, Proteo-Science Center, Ehime University, Toon, Ehime 791-0295 Japan; 7 Department of Behavioral and Community Health Sciences, School of Public Health, University of Pittsburgh, Pittsburgh, PA 15261 USA; 8 Department of Pathology and Microbiology, Faculty of Medicine and Health Sciences, Universiti Malaysia Sabah, 88400 Kota Kinabalu, Malaysia; 9 Mahidol Oxford Tropical Medicine Research Unit, Faculty of Tropical Medicine, Mahidol University, Bangkok, Thailand; 10 Centre for Tropical Medicine and Global Health, Nuffield Department of Medicine, University of Oxford, Oxford, UK; 11 School of Biodiversity, One Health and Veterinary Medicine, University of Glasgow, Glasgow, UK; 12 Faculty of Infectious and Tropical Diseases, London School of Hygiene and Tropical Medicine, London, UK; 13 Saw Swee Hock School of Public Health, National University of Singapore, Singapore, Singapore; 14 Department of Public Health Medicine, Faculty of Medicine and Health Sciences, Universiti Malaysia Sabah, 88400 Kota Kinabalu, Malaysia

**Keywords:** *Plasmodium knowlesi*, Zoonotic malaria, Community-based participatory research, Photovoice, Participatory visual method, Prevention, Rungus, Sabah, Malaysia

## Abstract

**Background:**

The control of *Plasmodium knowlesi* malaria remains challenging due to the presence of macaque monkeys and predominantly outdoor-biting *Anopheles* mosquitoes around human settlements. This study aims to explore the barriers and facilitators related to prevention of mosquito bites among rural communities living in Sabah, Malaysia using the participatory visual method, photovoice.

**Methods:**

From January through June 2022, 26 participants were recruited from four villages in Kudat, Sabah, using purposive sampling. Participants were male and female villagers, aged > 18 years old. After photovoice training in the villages, participants documented facilitators of and barriers related to avoiding mosquito bites using their own smartphone cameras, and provided narratives for their photos. Twelve Focus Group Discussions (FGDs) sessions in three rounds were held to share and discuss the photos, and to address challenges to the avoidance of mosquito bites. All discussions were conducted in the Sabah Malay dialect, and were video and audio recorded, transcribed, and analyzed using reflexive thematic analysis. The Ideation Model, a meta-theoretical model of behaviour change, underpinned this study.

**Results:**

The most common types of barriers identified by participants included (I) intrapersonal factors such as low perceived threat of malaria, (II) livelihood and lifestyle activities consisting of the local economy and socio-cultural activities, and (III) physical and social environment. The facilitators were categorized into (I) intrapersonal reasons, including having the opportunity to stay indoors, especially women who are housewives, (II) social support by the households, neaighbours and healthcare workers, and (III) support from healthcare services and malaria awareness program. Participants emphasized the importance of stakeholder's support in implementing feasible and affordable approaches to *P. knowlesi* malaria control.

**Conclusion:**

Results provided insights regarding the challenges to preventing *P. knowlesi* malaria in rural Kudat, Sabah. The participation of communities in research was valuable in expanding knowledge of local challenges and highlighting possible ways to overcome barriers. These findings may be used to improve strategies for zoonotic malaria control, which is critical for advancing social change and minimizing health disparities in malaria prevention.

**Supplementary Information:**

The online version contains supplementary material available at 10.1186/s12889-023-16173-x.

## Background

*Plasmodium knowlesi* malaria among humans continues to affect vulnerable communities living in rural areas in Southeast Asia [1]. Globally, while several regions have been declared malaria free status, such as China in 2021, and World Health Organization (WHO) European Region in 2015, the threat of *P. knowlesi* malaria remains [1]. Due to recent WHO policy changes, *P. knowlesi* is now the main barrier to malaria elimination in many Southeast Asia and Western Pacific countries [2]. In Malaysia alone, 2607 cases of *P. knowlesi *malaria were reported in 2020, despite successfully eliminating human malaria. The last reported case of indigenous human malaria in Malaysia was in the year 2017 [3]. The majority of *P. knowlesi* malaria cases were mild, but fatalities of patients diagnosed with this zoonotic malaria were occasionally reported [4]. Thus, the zoonotic malaria control strategy must be strengthened to address this growing public health threat.

The persistence of zoonotic malaria transmission is exacerbated by human interference with the ecology [5]. In Kudat, Sabah, Malaysia, deforestation activities are linked to zoonotic spillover of zoonotic malaria to humans [6], thus increasing the contact between *Anopheles *mosquitoes from the *Leucosphyrus* group and long tailed *Macaque fascicularis* or pig-tailed *M. nemenstrina* monkeys with humans. Studies have also identified a higher risk for *P. knowlesi* malaria among men, agricultural workers, and those who travel to the forest [7].

A growing body of evidence has shown that the underlying causes of zoonotic malaria infection are linked to its complex epidemiology [8]. In Malaysia, the core interventions against malaria are insecticide treated bed nets (ITNs) and indoor insecticide residual spraying (IRS), which are freely provided by the Ministry of Health (MOH) [9]. However, these indoor-based interventions are often ineffective due to the behavior of *Anopheles *vectors in these areas, which tend to be exophilic, exophagic and bite outdoors when night falls [10, 11]. Vulnerable communities are continuously at risk due to the presence of macaque monkeys and *Anopheles *mosquitoes [12, 13], and possible human-vector-human transmission [14]. Given these challenges, there is a need for additional research on human factors influencing exposure to *P. knowlesi* malaria [15]. Grigg et al.(2017) recommend studies on human behavior to understand further malaria exposure among at-risk communities [16]. While many malaria awareness programs have been conducted, there have yet to be in-depth studies on the community’s perspective done at the epicenter of human cases in Malaysian Borneo. It is critical to explore and understand the reasons for the challenges to avoid malaria or how current malaria control strategies can be improved to achieve WHO malaria elimination target by 2030 [17].

The WHO has recommended multisectoral approaches to control vector-borne diseases [18]. Community participation in research or health planning can be integrated with support from government and non-governmental partners to improve the effectiveness and appropriateness of public health program [19]. Moreover, malaria is a disease of poverty [20], and vulnerable communities are often the subject of study rather than key partners in research. As such, community-based participatory research (CBPR) could be a practical approach to involving the community in research [19]. CBPR involves research partnerships with community members toward understanding various local public health issues [21]. The influence of CBPR in addressing health inequities is growing [19].

The CBPR method applied in this study was photovoice. The method was developed by Wang and Burris in 1997 and was conducted with village women in Yunnan, China [22]. It has since been applied in a range of global contexts to explore complex health issues. Specifically related to malaria, Iskander (2017) explored different malaria beliefs and prevention approaches in the Philippines [23]. We built upon this work by exploring rural communities understanding of malaria, their prevention practices, and the challenges faced concerning its control.

There is a need to reimagine *P. knowlesi* malaria control, and to include the participation of the community in this process [17]. It is inadequately known whether and how persistence of malaria transmission is contributed by the mix of factors related to human behavior, the vectorial system, and/or ecological changes. Understanding potential barriers to malaria control, can contribute to designing effective and sustainable malaria interventions. 

## Methods

### Study aim

This photovoice study was conducted to explore and document the lived experiences and perspectives related to barriers and facilitators of preventing malaria among people living in rural villages exposed to *P. knowlesi* malaria in Kudat, Sabah, Malaysia.

### Study design

**Fig. 1:**
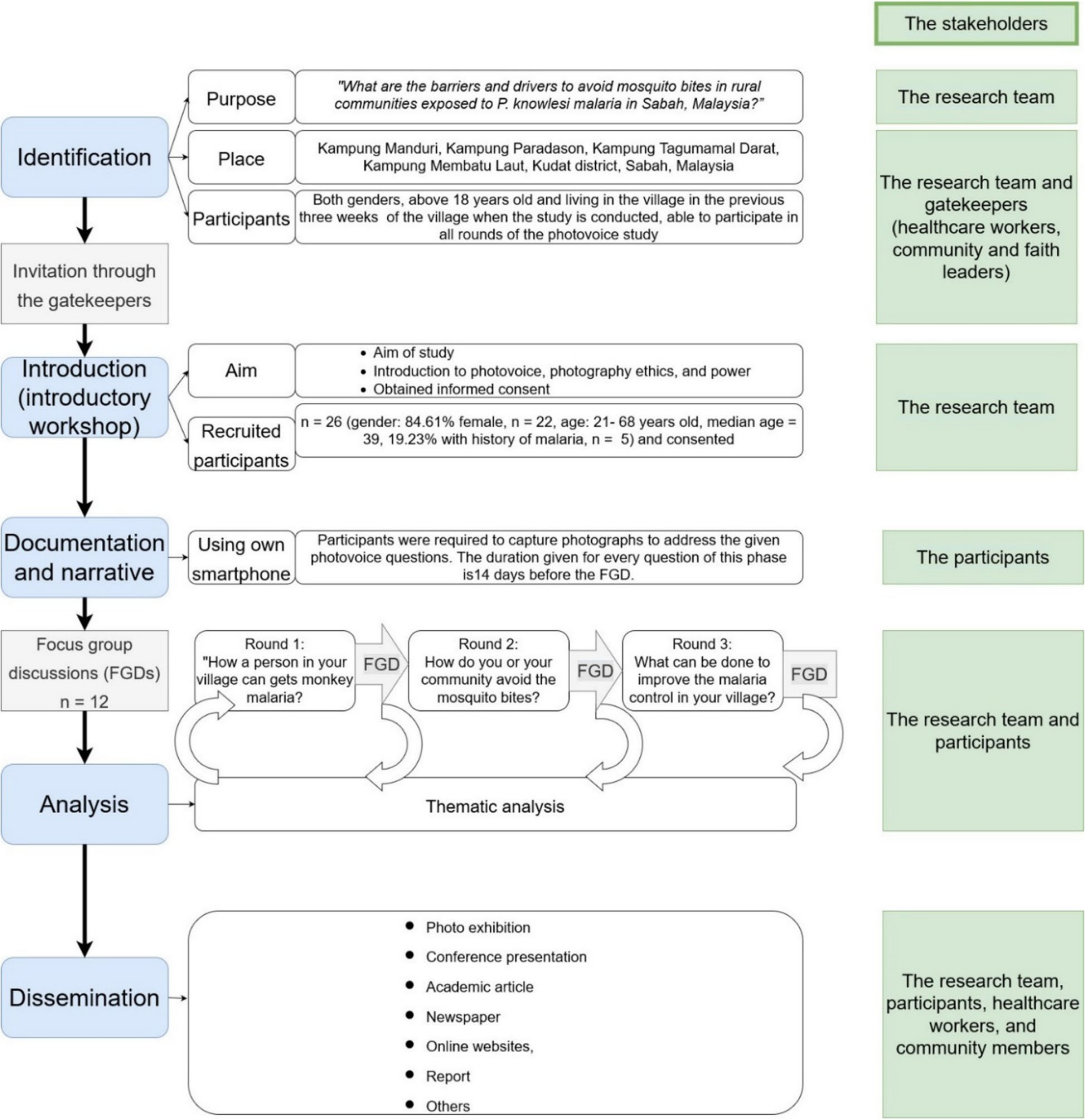
The photovoice study flowchart

A CBPR utilizing the photovoice methodology took place between 1 January and 31 June 2022. This approach invited participants to play an active role in documenting key issues, reflecting on those issues through critical dialogue, and raising their concerns to policymakers. Throughout the photovoice study, various stakeholders were engaged (Fig. 1. The photovoice study flowchart).

This study is part of a multimethod study to explore how communities living in areas exposed to *P. knowlesi* malaria avoid mosquito bites [24]. The study adapted the Ideation Model, a meta-theoretical model of behaviour change that highlights the influence of various social, environmental, and psychological factors on health behaviors [25, 26].

### Study sites

**Fig. 2:**
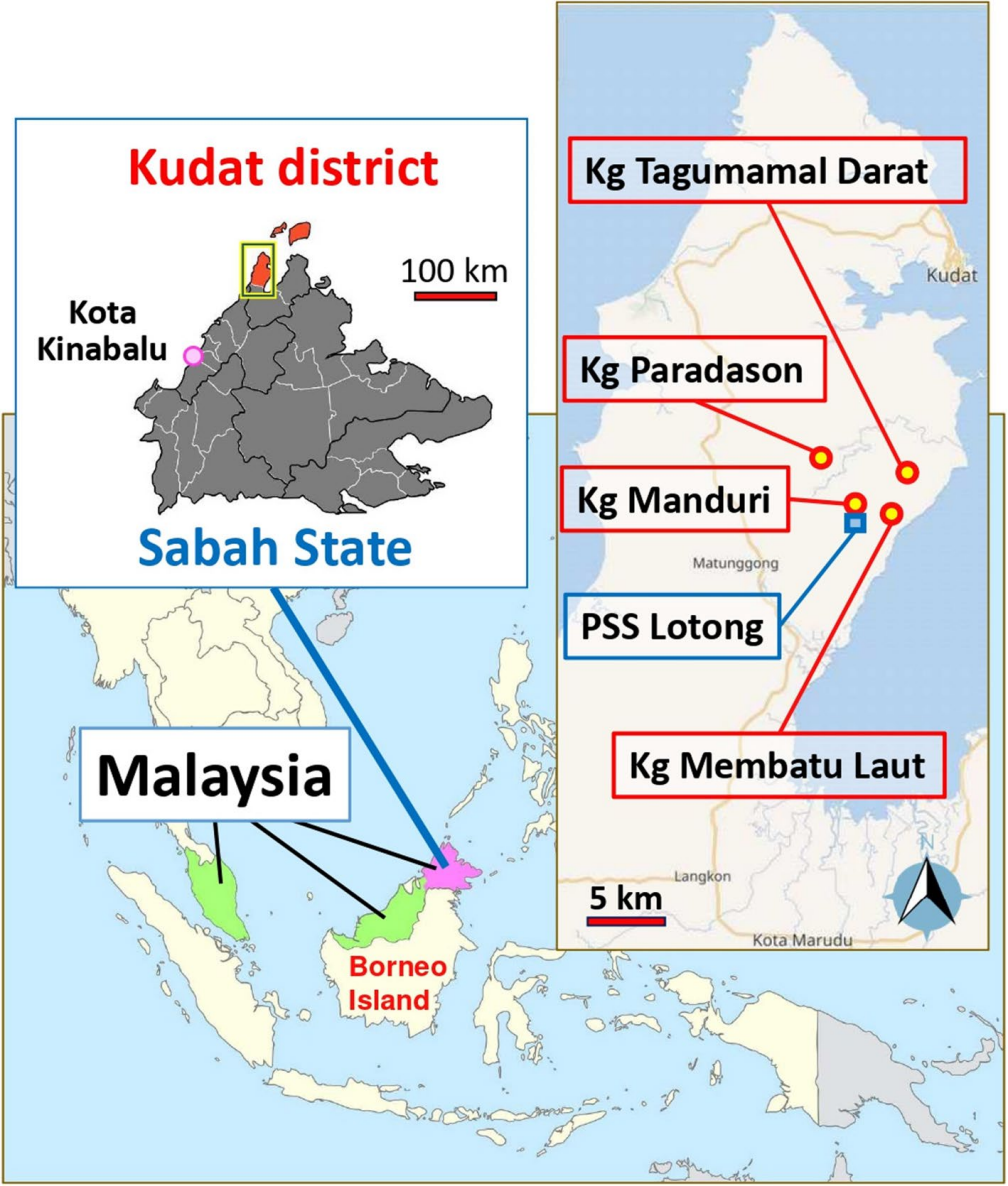
The study sites (Map data from Wikimedia and OpenStreetMap. Original Sabah map data is authored by Thomson Walt)

The study sites were located in Kudat, which is 150 km north of Kota Kinabalu, the capital city of Sabah, Malaysia [27]. The study was conducted in four villages: Kampung (Kg) Manduri, Kg. Membatu Laut, Kg. Paradason, and Kg. Tagumamal Darat. (Fig. 2. The study sites ("Map data from Wikimedia and OpenStreetMap. Original Sabah map data is authored by Thomson Walt). The study villages were selected based on data provided by the state health department as areas with a high incidence of *P. knowlesi* cases in 2020. All of the study sites were “red” localities defined as an area with incidences > 1 / 1000 per population based on the National Strategic Plan for Malaria Elimination [28]. These villages are under the surveillance of Pusat Sub Sektor (PSS) Lotong, a malaria subsector that handles routine malaria activities. The study area has been a focus of long-term research among various research groups [10, 29, 30] which could influence communities' perception of malaria. The region's primary language is Malay, but locals also speak indigenous languages (Rungus, Sungai or Dusun), or the Sabah Malay dialect.

The villages shared common environmental factors, with a wide distribution of plantations such as oil palm, rubber plantation, and forest-fringe areas. The main economic sectors in the district are centered around agriculture activities [31]. The typical climate of the study area is tropical, with a monsoon season from December to February each year. The temperature averages between 32 °C in the lowlands, and 21 °C in the highlands [10, 29].

Previous studies have reported the presence of the *P. knowlesi* malaria vector *Anopheles balabacensis* [10], macaque monkeys [13], and asymptomatic *P. knowlesi *cases in humans in the area [32]. Several studies have linked environmental changes (e.g., deforestation, commercial plantation, and small-scale farming) with the increasing incidence of cases in the population [6, 29].

### Community entry and recruitment

Before data collection, the study team informed and sought approval from the state health department, including relaying the study objectives, methods, and timeline. Upon arrival in the community, the study team met the healthcare workers who facilitated meetings with local gatekeepers [33]. A pamphlet was distributed to inform villagers about the study. Interested villagers contacted the researcher and were invited to an “photovoice introductory workshop”.

At the introductory workshop conducted for each village, the research team explained the aim of the study, research ethics, and collected written informed consent from those who showed interest in participating. Participants were informed of the pre-determined three rounds of photograph taking and Focus Group Discussions (FGDs). In each FGD session, participants were instructed to capture a maximum of five photos, focusing on various subjects such as humans, the environment, inanimate objects, or other items that helped to tell their stories about facilitators and barriers to malaria prevention. Photographs of minors (children below 18 years old) were not allowed due to ethical considerations. Participants were also informed of the need to get consent from people who appeared in photographs. This was critical because of ethical considerations around the use of photography, in which participants and community members may be identifiable [34]. No other guidance was provided on how photographs should be taken, as the research team wanted to capture the unbiased perspective from the participants. This also helped to ensure a naturalistic approach to inquiry [21]. At the workshop, participants were also informed about the proposed dissemination plan, and how the study’s photographs, narratives, and findings would be shared with policymakers.

### Study participants

Purposive sampling was employed for this photovoice study. Previous studies have identified adults above 15 years old as a high-risk group for *P. knowlesi* malaria [16] and both genders are at risk of infection [16, 32]. In this study, eligible participants were adult villagers (> 18 years old), male and female, living in the village (defined as staying in the village for the previous three weeks, at a minimum) and who consented to participate. The study adapted the duration Grigg et al. used to recruit the village residents as study participants by specifically including individuals who resided in the study area in the last three weeks [16]. The study excluded community members who could not attend the discussion after two rounds. Due to the COVID-19 pandemic, only fully vaccinated participants were allowed to participate in the study; however, due to the high level of vaccination in the study population, this had minimal bias on the study recruitment procedures. All participants and researchers wore masks during the sessions to avoid close contact-disease transmission. Thirty-two participants attended the first introduction session (eight participants for each village). Previous studies recommended five to eight participants per focus group discussion (FGD) for effective group dynamics [35]. However, six participants had to withdraw for personal reasons such as hospital admission that required further follow-ups (*n* = 2), migrating to other areas (*n* = 1), time limitations, and responsibilities related to childcare (*n* = 3). In total, 26 participants completed all the photovoice discussions from four villages (Kampung Manduri, *n* = 6, Kampung Paradason, *n* = 6, Kampung Tagumamal Darat, *n* = 8, and Kampung Membatu Laut, *n* = 6). The characteristic of participants in more detail [see Additional File 1].

### Research team and partners

The study was led by N.A.N, supported by all co-authors, and data was collected in partnership with several local and international partners. Supervisory support was provided by M.R.H, R.H.and M.S.J. (public health specialists). Qualitative advice was further supported by P.Y.P.L, with expertise in medical anthropology. Additional support was provided by A.M., R.C., S.S., B.A., and K.F. (malaria experts), S.B. (participatory and arts-based research expert). International photovoice experts guided the photovoice study (G.B., A.C.M., N.W.). The research team and experts had online meetings three months before the study to ensure the research team was well-versed in the ethics and procedure of photovoice study. Informal discussions were done with these experts throughout the study.

### Data collection

This photovoice study adapted the phases proposed by Latz et al. [34]. All questions were drafted, discussed with coauthors, and piloted with four local Sabahan colleagues to ensure the prompts were understandable [35]. The Sabahan colleagues who assisted in piloting the questions shared similar characteristics with the study participants. Specifically, they have first-hand experience living in rural villages and/or have relatives living in malaria-exposed areas. This similarity in experiences enhances the relevance and validity of the questions used in the study. The transition of questions from draft to questions used during the sessions are described in Additional File 2]. While the piloted questions helped to ensure consistency, the researchers also adopted flexibility, considering the community-based nature of this study. During the FGDs, participants were asked to explain the captured images using the SHOWeD questions [34], which stand for:

S: What can we “See” from this photograph?

H: What is really “Happening” here?

O: How does this relate with “Our (your)” life?

W: “Why” does this photograph relates to our inquiry, such as barriers to malaria prevention?

D: What can be “Done” about the issues or challenges highlighted in this photograph?

There were three rounds of photography and FGDs for each village. The first round focused on local knowledge of malaria causation [36]. The second round aimed to understand the challenges of avoiding malaria and their preventive practices. The final round allowed participants to share their recommendations. During the third round, member checking was conducted to confirm the key points (preliminary themes) and avoid misinterpreting the participants’ perspectives. The duration of each FGD session ranged from 60 to 90 min. Prior to the discussion, participants themselves selected the photographs. There was an interval of 20 to 30 days between each FGD round.

Local expert translators in the Rungus language were recruited to assist in the FGD as the majority of participants were of Rungus ethnicity. All the discussions during the FGDs were conducted in Sabah Malay dialect in private locations accessible to all participants, such as the village hall, and audio recorded. Snacks were provided for all FGDs. Each participant received RM10.00 (USD 2.20) of phone credit for each round of FGDs, sufficient for uploading, downloading, and continuing contact with the research team.

### Data analysis

Data analysis was an iterative process that began during data collection. At the end of each FGD, the moderator reflected on the discussion and shared her understanding, including key highlights with the participants. Before concluding, the moderator asked the participants, “*Do I understand it correctly*?" or "*Is there anything that I missed?”*. Participants were allowed to correct the misinterpretation of the moderator. This iterative approach facilitated the member-checking process to ensure the trustworthiness (credibility) of the findings [37].

The reflexive thematic analysis described by Braun and Clark was used to guide the study findings [38]. All audio recordings were transcribed by N.A.N., using Sabah Malay language, as similarly as they were recorded, to allow familiarization with the dataset. A deductive-inductive approach was used for the coding process, whereby patterns and meanings were identified, compared, and interpretated across the datasets [38]. The deductive process was facilitated by the themes generated from a previous Delphi study conducted with malaria experts, which provided a framework for organizing and structuring the initial codes [39])(Additional File 3. Thematic analysis with deductive-inductive approach). Through the researcher’s subjectivity and critical reflection [38], N.A.N generated the codes, and confirmed the findings with the participants. In addition, during the iterative FGDs, any new codes and themes expressed by the participants were discussed to avoid misinterpretation by the moderator. To increase the richness of the findings, N.A.N, P.Y.P.L, M.R.H and R.H collectively reviewed and defined the theme. When finalizing the study findings, related quotes were translated into English by N.A.N, M.R.H and P.Y.P.L.

All transcripts were coded in ATLAS.ti 9.1.7. software, which facilitated systematic documentation and presentation of the data. The documents within ATLAS.ti were password protected to ensure the access of data to the research team only.

### Dissemination

**Fig. 3:**
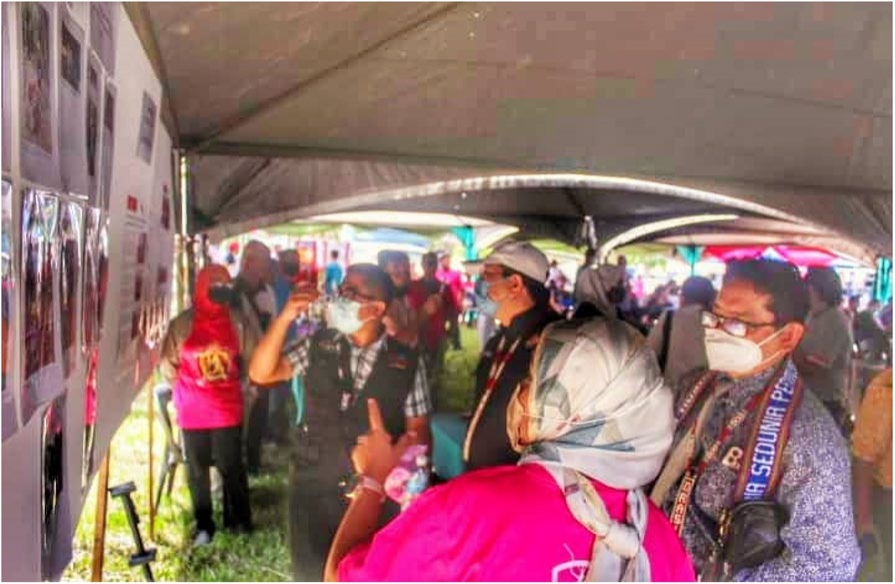
Photovoice exhibition during World Malaria Day year 2022 in Kudat, Sabah, Malaysia

The dissemination platforms used to share the findings ranged from photo exhibitions, academic manuscripts, conferences, newspaper articles, and online websites [40, 41]. The participants agreed to share the photographs publicly and credited with anonymous identifiers. Several participants allowed their names to be included as identifiers. During the photo exhibition at the village, the research team invited officers from the district health office, community leaders and community members (villagers). Academicians from local universities were invited to share their insights related to malaria research and proposed opinions on future studies. The photovoice project was invited to the Sabah state World Malaria Day 2022, where policymakers (e.g. MOH, district health officers, politicians) and stakeholders were invited by the Kudat district health office (Fig. 3).

## Results

A total of 26 villagers participated in the study; 22 participants (84.63%) were female, and the participants ranged between 21 and 68 years old (median age 39). Seven participants (26.92%) had a history (or histories) of malaria over 12–24 months before the study was conducted. Almost 90% of the participants are from the Rungus ethnicity. According to the participants, majority of the inhabitants of the communities were from the Rungus ethnicity. The minorities were from the Dusun, Sungai, and other ethnicities. Many had inter-ethnic marriages. Many were Christians (Protestants), and a minority were Muslims. According to the participants, one or two people in the neighboring villages were Pagans. All participants completed the three data collection rounds. This study did not collect detail of income and expenditure as it was influenced by season (e.g. raining), and availability of the work that might differ due to various reasons. In this study, the measurement of income used other proxy measures such as housing condition household appliances and amenities [27].

Over the six months of study (January to June 2022), a total of 215 photographs were captured. On average, each participant took two to three photographs each round. At the time of the study, all participants reported that mosquito bites transmit malaria, and the *'kuman malaria*' (parasite) originated from monkeys. All had heard about preventive measures through various resources: healthcare workers, communication in the village, health program and through previous research teams in the area. Everyone had been given ITNs and IRS was done in their villages. 

The results are presented by key sub-themes of barriers and facilitators to mosquito bite prevention and illustrated through photographs and direct quotes from study participants.

### Barriers to avoiding mosquito bites

#### Theme 1: Intrapersonal factors

##### Subtheme 1: Low perceived threat of malaria

According to the participants, despite having a good understanding of the causes of monkey malaria, some individuals were “unaware” or chose to ignore the threat of these mosquito bites. Even individuals with a history of malaria were not necessarily motivated to take preventive actions against mosquito bites. Participants explained the potential consequences of not listening to advice from trusted individuals. For example, one participant shared an instance where her husband neglected to wear full-sleeved clothing as a precautionary measure. She explained, “*People do not put on anything to avoid mosquito bites. It is common here. People wear short pants and shirts* “[Female, 55 years old, Kg Manduri].

The participants described how some people viewed themselves as too strong or “lucky” to be spared from the malaria infection.

“*They understand how a person could get malaria. They understand the risk of the mosquito bites… but there are those who said ‘Ah! I am resistant to the infection [also malaria]!’ Some believe they are lucky and they would not get malaria. Like my husband, he has a history of malaria. If they already mentioned such, nah – that’s it. I think the person wants to show that he or she is strong and can avoid the infection.*” [Female, 33 years old Kg Membatu Laut].

##### Subtheme 2: Low perceived response efficacy to use vector control measures to avoid mosquitoes

Participants reported low perceived response efficacy for the current malaria control measures, such as sleeping under bednets, using mosquito repellents indoors or outdoors, and wearing protective clothing to avoid mosquito bites. Participants perceived the available interventions as ineffective for preventing mosquito bites. For example, the repellents that produce smoke do not entirely protect them, as the smoke follows the wind. The participants described that the current items are inadequate to protect them from mosquitoes. They said some people used these items, but were still infected with malaria.

“*Nearby my house, there is a pond and many mosquitoes there. They (the healthcare workers), caught the mosquitoes there. Afterwards I heard one of them was infected with malaria. I have been infected many times, more than 20 times [...]. When I went hunting animals or walking to my farm, I seldom used any measures to avoid mosquitoes. I did wear long pants and long sleeved shirt, but in my opinion, it did not fully protect me. They (the mosquitoes) can bite my neck and face. I always tell them (the healthcare workers), please find ways to kill the mosquito eggs.*” [Male, 62 years old, Kg Tagumamal Darat]

There are other reasons for poor self-efficacy in using the prevention items. For example, usage discomfort, feasibility, inconvenience, and movement restrictions.

“*Activities like breeding the chicken is a daily activity. People do it in the morning and evening, just around their house. Undoubtedly, they are exposed to mosquito bites. People hardly, or do not, use anything to prevent it. Why would someone bother to put on long pants or shirts if the chicken pen is just outside their house? They are used to it [the mosquito presence].*” [Female, 45 years old, Kg Membatu Laut]

Participants shared their concern about the large holes in bednets, which enabled the mosquito to fly in. Holes are defined as a tear or opening that a finger could fit through.

“*The mosquitoes can fly through the bednet holes. The holes are pretty big (while pointing to the blue nets). The bednets, too, it is hot. When I sleep under it, it is hot. We do not use fans here. There is no electricity (while pointing to the green bednet). There is no airflow if I sleep under the green bednet.”* [Female, 40 years old, Kg Paradason]

#### Theme 2: Livelihood and lifestyle activities

##### Subtheme 1: Livelihood and local economy

According to the participants, agricultural activities are the primary source of income and food sources. Communities are engaged in agricultural activities such as small-scale farming, rubber tapping and working in the oil palm, fishing, collecting non-timber forest products, and driving heavy machines for income. Participants explained that people in their village use traditional methods for cultivation, often with bare hands and traditional utensils.

Both male and female villagers generally conduct farm work as it brings more cash return to the household. In these settings, the low socioeconomic status impacts the purchase of malaria intervention measures (e.g., mosquito coils), as villages must weigh the need to purchase food, telephone prepaid, and other materials for life.

Many female participants shared activities that possibly exposed their husbands to malaria. Their livelihoods include fishing, breeding chickens, rubber tapping, and others. A breadwinner (and their partner) can have multiple types of work to support their family. Women sometimes follow their husbands; some stay home and conduct household chores.

*“I do many jobs to support my family […] I do rubber tapping, pluck the coconuts, farming, planting […] cut the grass at my farm […]. When mosquitoes are around, I do like this [pretending to swat the mosquitoes using a cloth].”* [Female, 55 years old Kg Paradason].

**Fig. 4:**
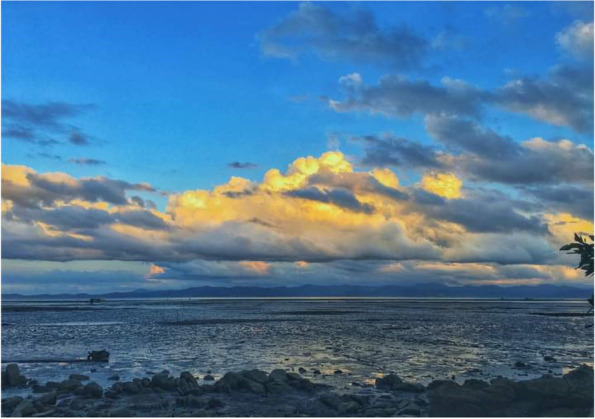
“*This is the beach during the sunset, around 6 o’clock. I took the picture before I returned to my home after I caught the snails and clams at the beach*.” [Female, 27 years old, Kg Tagumamal Darat]

Fishing, for example, exposes all age groups to malaria. Participants expressed that wearing long pants, long shirts, and protecting their heads to avoid mosquitoes is not feasible for fishing activities. Short pants and shirts are more comfortable, and the attire makes them less wet (if the water “absorbs in their clothing”). While they understand the risk of malaria, wearing mosquito repellents is not feasible, as the body will be wet during these activities. Furthermore, fishing at sea does not have a fixed time as it depends on the water tides (Fig. 4). Those who go fishing at the river and ponds prefer nighttime, as the small fish and prawns come out when its dark. At this time, they are particularly vulnerable to mosquito bites.

A participant said she had a history of malaria in 2020, possibly from fishing. However,without alternatives for the livelihood, she continues to conduct activities outside her house (e.g., farming and fishing). Fishing activities sometimes involved the entire family members.

“*People who were infected with malaria are those who go to the farm and rivers at night. This includes children, nine to eight years old, both adults, men and women. Children, around the age of nine to ten years old, they go fishing, from morning ‘til night time. That is how they can get malaria.*” [Female, 55 years old, Kg Manduri]

##### Subtheme 2: Socio and cultural activities, celebrations and gatherings

For some participants, their morning activities started as early as 4 o’clock in the morning. It is rare for people in the community to wake up after 6 o’clock in the morning. Typically, the wife (woman) wakes up the children, prepares them for school, and prepares breakfast for the family. Some families have to walk some distance from the house to clean their bodies and utensils. Some wives, afterward, will go to the rubber farm.

**Fig. 5:**
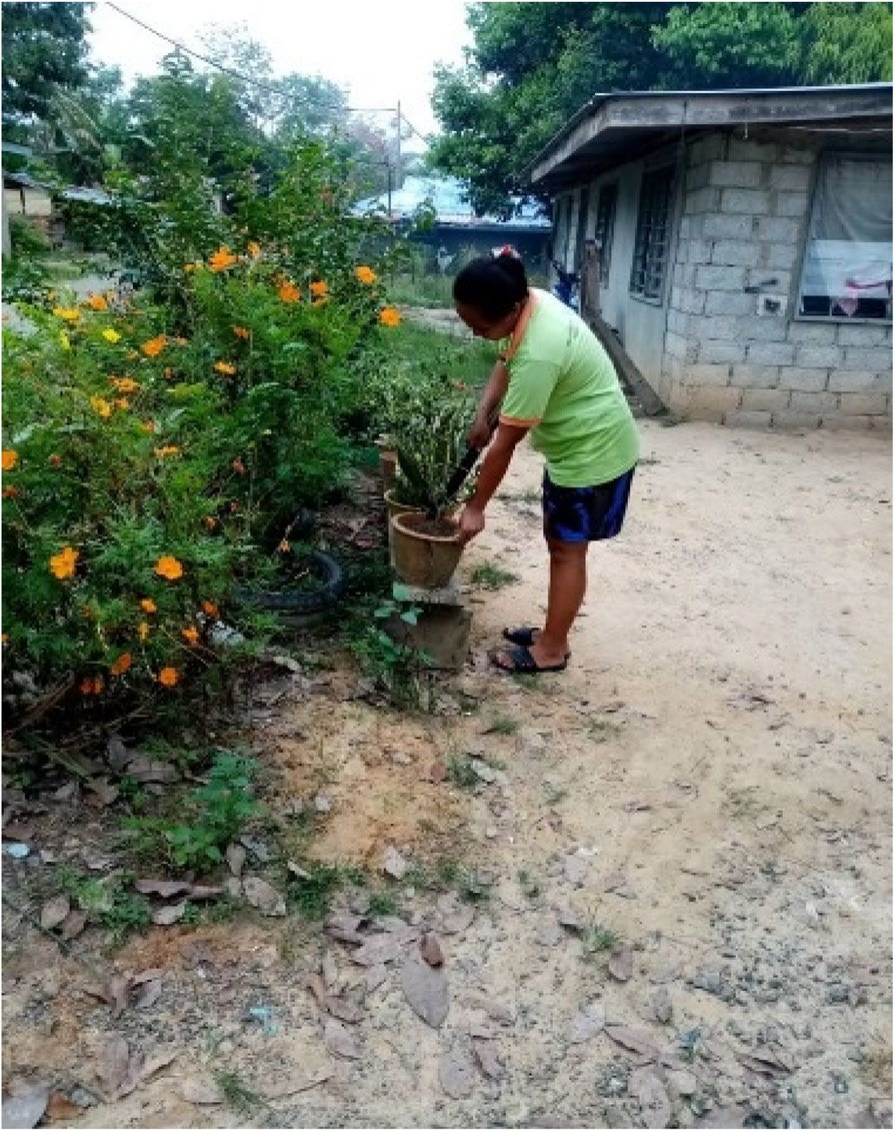
“*Doing gardening and watering the plants around the house.*” [Female, 33 years old, Kg Manduri]

More than half of the participants were housewives, who conduct multiple activities throughout the day, even from late evening until midnight. While spending time with their children (e.g., doing schoolwork, reading, writing, playing in the house), they also do routine domestic activities (e.g., cooking, cleaning the house, gardening). Additionally, they relax and/or gather with relatives, neighbours, or their friends, and look after the chickens and pigs outside their house. Women who do gardening prefer to wear simple and light clothing (e.g., short sleeved shirts and pants), as it is more comfortable. Gardening is done in the morning, or late evening, as it is less hot and humid. For example, cleaning the house's surrounding area (e.g., cutting grass) is done in the late afternoon to almost night to avoid the hot sun (Fig. 5).

“*Usually, I will clean the areas outside my house in the evening […] I water the plants. I wear normal clothing.*” [Female, 34 years old, Kg Manduri]. Normal clothing is described as the everyday clothes they wear at home, such as t-shirts, pants, and sarongs for women.

Participants described the importance of outdoor activities for maintaining a healthy lifestyle, despite knowing the risk of malaria. One of the female participants adopted jogging as one of her hobbies to keep her body fit and lean. All ages, and both genders in the village engaged in sports or other healthy activities until late at night. People who play badminton and sepak takraw (English; kick volleyball) play the game in the village hall, with open doors and windows. One female participant from Kg Manduri, played badminton too and accompanied her three children to the hall:

*“I go there and play badminton, usually until 6 o’clock in the evening, and watch my kids playing their sports there. It could last until midnight. Even my husband plays sports with his friends until late at night.*” [Female, 32 years old, Kg Manduri]

In significant events such as a village wedding, communities perform karaoke until late at night. Other social activities, such as drinking ("*aramaiti*") and socializing in the evening are also common. These activities are social norms in the villages. During social events, women will usually stay inside the house, while men commonly sit and talk outside the house, wearing shirts and pants. Thus, these activities expose men to higher malaria exposure compared to women. Participants reflected upon the possible reason for getting malaria, stating that it was likely due to engaging in outdoor activities until late at night, despite knowing that mosquito bites that can cause malaria.

*“In our culture, relaxing, gathering, and drinking alcoholic drinks, or as we call it aramaiti, is done anywhere. It could make a person forget, and become unaware of the risk of malaria. When the person drinks too much, until he is drunk, he or she could sleep anywhere. Even outside their house or under the drinking table. It happens here, around the villages. It is common here, a norm, this aramaiti.*” [Female, 59 years old, Kg Membatu Laut]

**Fig. 6:**
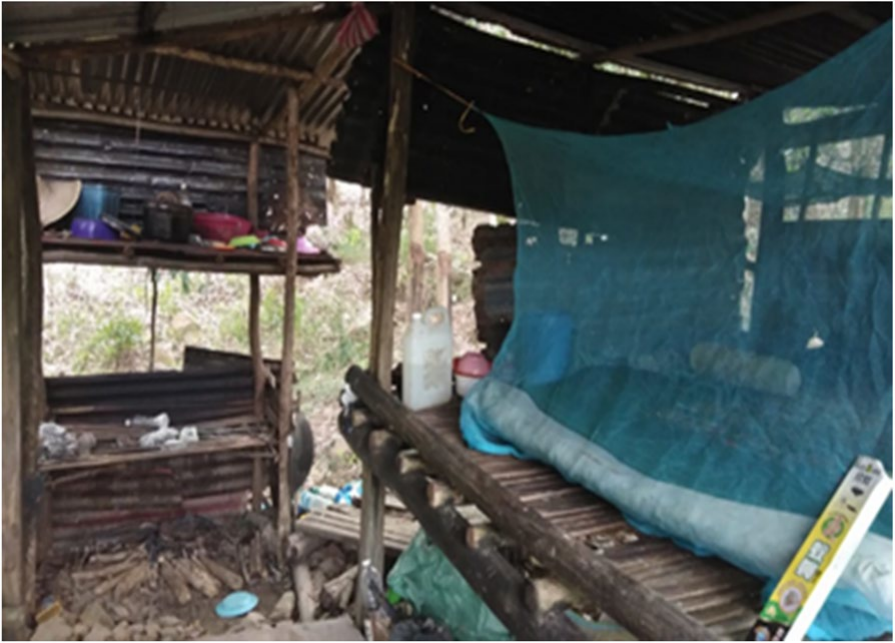
“*The farmers usually will sleep in the hut at the farm. They use different ways to avoid mosquitoes, such as sleeping under the bednet, burning the leaves and grasses, and closing the water containers. But some just do not practice it.*” [Female, 49 years old, Kg Tagumamal Darat]

Participants had other local views about the night-time activities in the village. During the dark hours (nighttime), the villagers did activities such as rubber tapping, honey hunting, or even hunting for wild animals. If the farm is far from their home, villagers will sleep in “*sulap*” (English: hut) located in the forest, or plantation/farm that is surrounded by the forest (Fig. 6). Participants explained, rubber tapping activity is preferably done at night as it could produce more latex. However, some villagers have their own preference which depended on the availability to go to their farm.

For those who search for wild honey, their reason was that during the dark hours, they can protect themselves from the bees in the darkness. By lighting up a fire nearby, these hunters will take the honey from the wild beehive, while the fire will attract the bees. They have a higher risk of getting stung by bees when they search for wild honey during the day.

Forests surround the villages, and many wild animals, such as deer and wild boars, are hunted. Villagers who hunt do so during nighttime to early morning as this is when the wild animals roam around. However, during hunting, hunters do not use mosquito repellents and are unable to carry heavy loads. They need to be mobile and move fast. Hunters need to blend with the environment; thus, using any items that release odors (e.g., mosquito repellents) may trigger the animal sense to avoid the area.

“*When a person goes hunting for wild animals, they will not wear any repellents. The animals will run. They could smell the insect repellent from afar. The hunting activity is done at night time. Anyone with no job in the morning will go hunting […] Also, it is their hobby.*” [Female, 52 years old Kg Membatu Laut].

##### Subtheme 3: Weather and outdoor activities

**Fig. 7:**
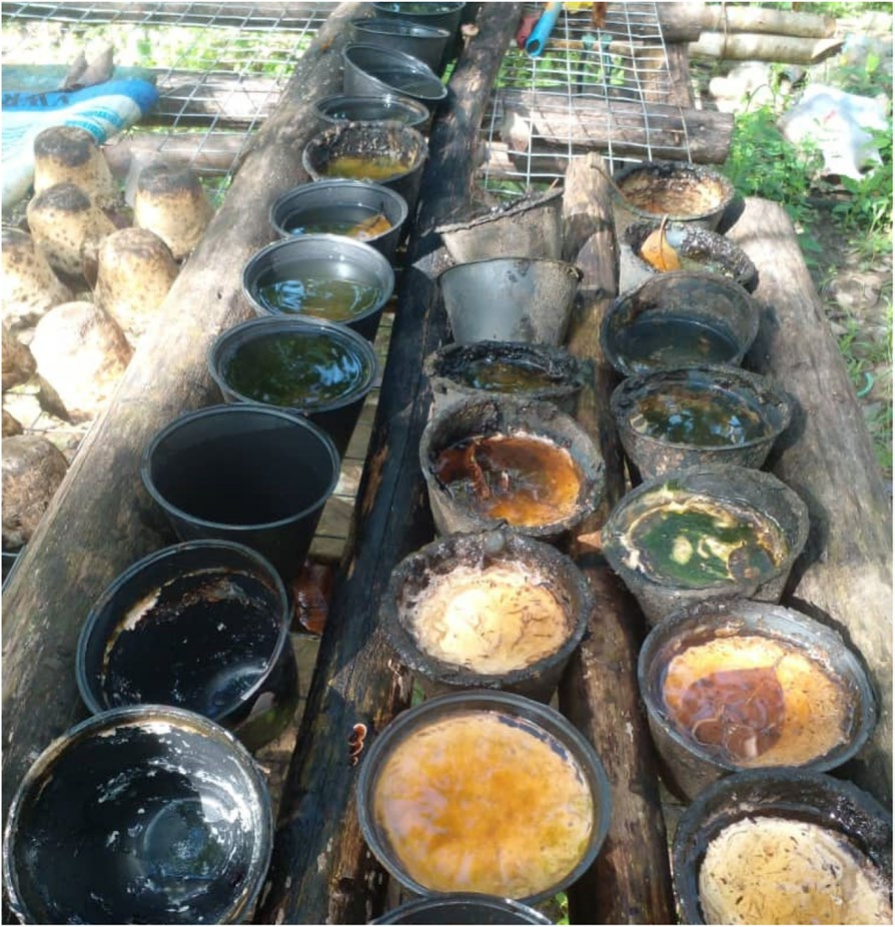
“*During the rainy season, the water will be stagnant in the containers with mosquito larvae. This container has been left in this area during the rainy season. This is a risk as the mosquito could breed, but this activity is for our livelihood*.” [Male, 46 years old, Kg. Tagumamal Darat]

Many activities in the villages are heavily influenced by the season (rain or sunny) and timing of day (morning vs nighttime). Rubber tappers' activities are heavily dependent on the weather; during non-rainy season, it becomes a daily activity. During rainy season, the rubber tapping activity is less frequently done. Therefore, the rain water, which is collected in the latex cups and other items, become areas for mosquito breeding. These items generate temporary aquatic habitats for mosquito larvae (Fig. 7).

#### Theme 3: The environment

**Fig. 8:**
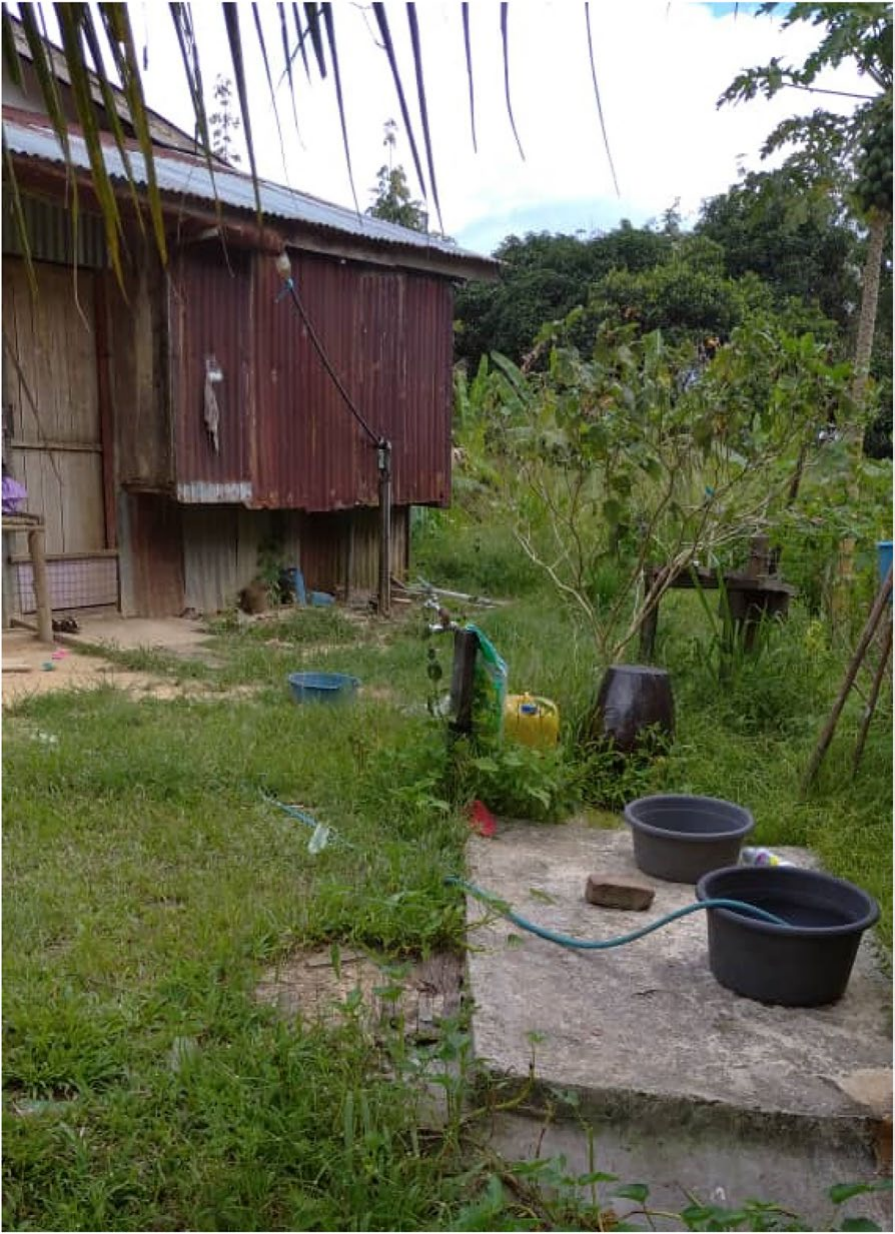
“*People can get mosquito bites outside the house. This is because there are bushes and long grasses that are not cut. There are containers too. We need to put it upside down to avoid the mosquitoes from breeding in these areas.*” [Female, 29 years old, Kg Manduri]

According to the participants, their risk of mosquito exposure is also influenced by the surrounding environment. This include not only the nearby forest but also the farms, plantation areas, and the conditions around their houses (Fig. 8).

##### Subtheme 1: The physical environment

The participants described their villages’ environment as attracting the presence of long-tailed monkeys and mosquitoes. Participants also realized that more monkeys were coming nearby and into their home. The mosquito was also noticed to come earlier to bite, even before dark. According to one participant in Kg Manduri, there are no forest reserves in the village due to human interference in the village area. Participants identified human interference activities, like logging, timber activities, and conventional plantation (e.g. Acacia trees), disrupting the villages' natural environment including the monkeys' habitat. In addition, small-scale farming and plantation such as rubber trees in the village also played a role in these contexts. Some of the activities were described to be resulted from previous interference of non-local developers on the local lands.

“*There are monkeys and mosquitoes here, everywhere […] at the river, rubber trees area, paddy fields.*“[Female, 40 years old, Kg Manduri].

**Fig. 9:**
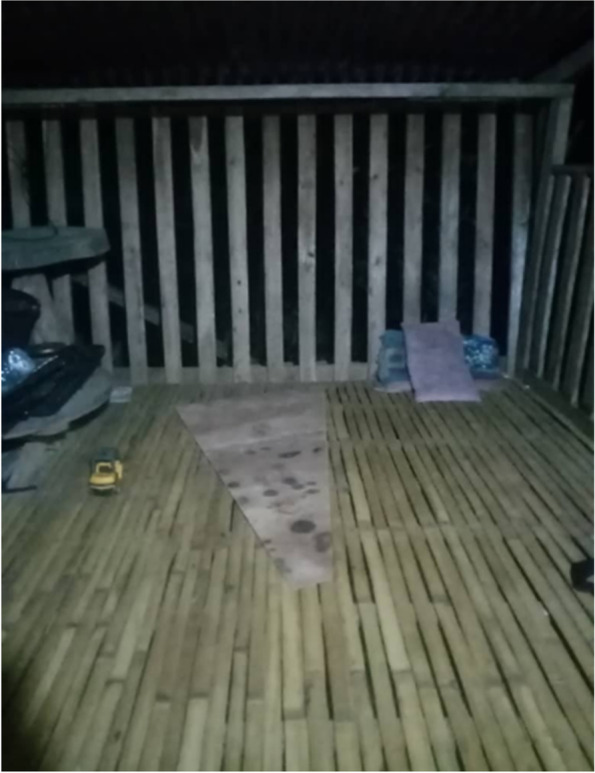
“*Relaxing, talking, and eating at the “tingkang” before going to sleep*.” [Female, 40 years old, Kg Manduri]

Participants explained that many of the villagers’ houses were built with bamboo, rattan, plywood, and forest materials, providing a route for mosquitoes to enter. There are holes in the walls, ceilings, and floors. They are also a unique local structure in the houses called “*tingkang*”. “*Tingkang*” is defined as areas like verandah, but built with bamboo/ratan and usually served as a place for gathering and relaxing in the house. “*Tingkang*” walls are built, similar to a picket fence, to provide good airflow during these activities (Fig. 9). The local bathroom is built separately from the house’s main structure, due to the unavailability of an adequately treated water supply built for them.

“*My house was built using the “kayu bulat” (English: timber product]. There are cracks (gaps between the flooring planks), and there are many mosquitoes indoors. Anytime, in the morning, night […]. There are a lot of mosquitoes.”* [Female, 38 years old, Kg Manduri]

According to the participants, the presence of people with a low perceived threat towards hygeine puts other people at risk of malaria. For example, participants explained that garbage around the villages not only made the area dirty but also promoted breeding grounds for mosquitoes. In the villages, there were no garbage collection facilities. Thus, garbage were either burned or dumped around the villages.

##### Subtheme 2: The social environment

The communities from all four villages were small in size and built on strong family ties where everyone knows one another. Cultural and social gatherings is a norm, and regularly conducted outdoors until night-time. Communities celebrated religious ceremonies such as the *Keamatan* festival, Christmas, and *Hari Raya*. The strong cultural gatherings are also held for funeral, mourning and remembering the dead, which could last for 30 days. During these cultural celebration and activities, there are minimal useage of malaria interventions. Participants described eventhough they live in a low socio-economic lifestyle, they tried to put on their best effort to survive the daily life.

However, participants shared their frustration about water-source issues. Even though there were piped water-systems, they did not work continuously to provide treated water sources to communities on a daily basis. In one of the study villages, participants stated that there is no piped water system. Communities are “forced” to go to the river to get water. Some participants practice keeping water in water storage (e.g., in basins). Participants explained that they needed to go to the river (despite knowing the risk of malaria), as that is the only water source. Some villagers who could afford to pay for the water gravity system have water supplied to their homes. Nevertheless, even though gravity water was available, the supply was not continuous. The issues with access to treated water supply have been ongoing since the existence of the village.

“*It has been almost two weeks without water supplied to our house, can you imagine? I take my shower and clean my body at the river. We have had no water until now, this is very irritating, and I am mad!”* [Female, 45 years old, Kg. Membatu Laut].

Housewives who had to do domestic chores (e.g. washing their clothes) need to go to the river.

“*In the village, there was always a problem with the water supply [the piped water]. We have other sources like gravity water, but it depends on whether the water has enough pressure to supply to our houses. In my house, the pressure is not very strong. We could go to our relative's house, which is nearby [around 200m]. But, I prefer to go to the river, because it’s easier for me to do my chores like washing clothing. I have many clothes to wash [calmly speaking][…]I will go to the river around 5 or 5:30 in the evening. It is already nearing dark by then […], and when I have completed washing the clothes, I clean my body and return home in the darkness of night* [Female, 40 years old, Kg Paradason].

In the village, participants reported that poor or no internet connection made the community vulnerable to mosquito bites/malaria. They need to find areas with a good internet connection to use their telephone or any electronic items that require an internet connection. These areas were situated in the village themselves (with forests, plantations, etc.). During the COVID-19 pandemic, school children had to risk their health by going outdoors and seeking internet lines to attend classes. Teenagers, even adults, were often seen “gathering” in areas where the internet connection was strong*.*

*“They play games on their phone outside their house [laughing]. They go outside to find a good line connection for their phone. The kids went outside to find internet line […] from midnight until the sunrise. They either ignore or are resistant to the mosquito bites!” [Female, 33 years old, Kg Membatu Laut]*

**Fig. 10:**
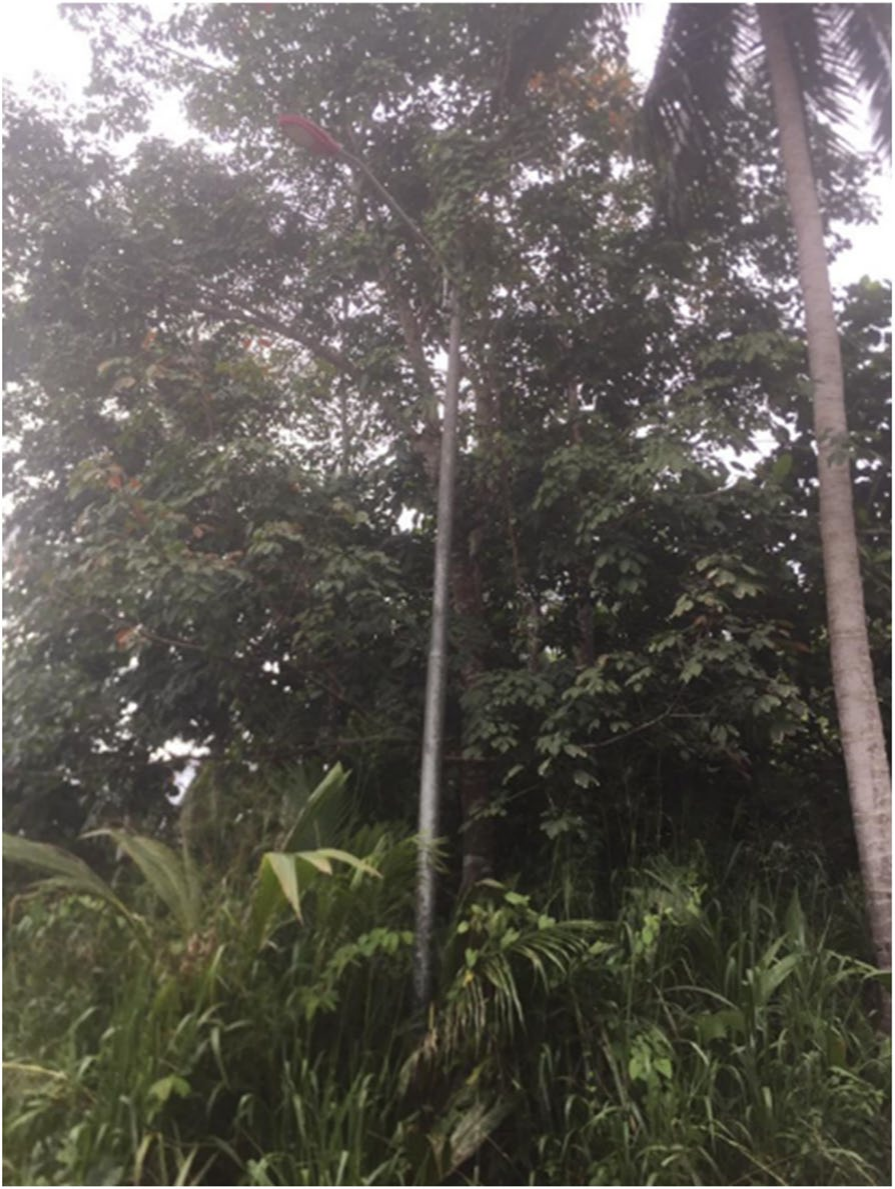
“*This lamp post has been here for a long time, but there is still no electricity. There is no electricity in our house too. We only use candles. It always feels like nighttime here. Moreover, mosquitoes like this condition. We are always at risk of getting mosquito bites [malaria].”* [Male, 32 years old, Kg Paradason]

In one of the villages, where there was no electricity supply, ‘*polita*’ (Eng: oil lamp) became an alternative light-source. They described the houses as often “gloomy” and “dark”, which attracts mosquitoes. Participants raised concern on the electricity issue despite “modernization” in other areas. They felt their lives still resembled the “*zaman dulu-dulu*” (English: previous era, prior to the existence of electricity). Even though there are electric lamp posts that have been put up around the village, there was still no electricity. They mentioned about their inaffordability to apply for the lamp post as one post alone will cost them more than RM1000 (USD 212.68). To them, there were other competing essentials that they could use their money for (e.g. food, etc.) (Fig. 10).

“*The issue is […] with community leaders that should lend a hand and support us [the local development]. There are areas with electricity […] there are also areas without electricity in the village. Those with electricity, they applied for it. They paid for it. However, for us, we do not have the money. The wiring itself is expensive. We could not afford to apply for it. One electric post cost around thousandths ringgit, and that excludes the wiring. We always live in darkness here”. [*Female, 40 years old, Kg Paradason]

### Facilitators to avoid mosquito bites

#### Theme 1: Intrapersonal factors

The ability of a person to practice preventive methods to avoid mosquito bites offers protection and lowers the risk of malaria. 

##### Subtheme 1: Perceived threat and awareness of malaria 

Across all study sites, participants described malaria as a threat. Participants understood the disease's causation and transmission and that they were always vulnerable to malaria. They were also aware of the condition and malaria risk in the villages. These factors strongly influenced their decision to avoid mosquito bites. For example, the limited access to treated water supply made them shower and clean their body earlier, around 3 to 4 o’clock in the afternoon. Being aware that mosquito bites when the day gets dark and the risk of malaria due to environmental condition, participants wore protective clothing (long shirts and pants, however they infrequently cover the head). Although they pointed out the importance of wearing protective clothing, they understood that it had limitations, as mosquitoes can bite at areas that are not covered by the cloth, such as the hands, fingers, the head and neck. Even though they initially wore clothing, due to high temperature and sweating, some parts of the clothing were lifted to allow for airflow.

Participants emphasized the need to avoid stagnant water in containers, especially after the rain. Knowing the risk of mosquito breeding in the containers motivated them to clean the containers and/or flower vases. *“If you passed by any containers, where there is water present, you just pour the water out […] if the containers are not in use (latex cups, vases, and others) […] just turned them over to avoid them from collecting water* [Female, 42 years old, Kg Membatu Laut].

Participants also shared their experiences of having family members or households previously diagnosed with malaria as a motivating factor to perceive the threat of the infection and avoid malaria. They became more aware of the threat, especially when their spouses were infected with malaria. Having experienced household members who had malaria motivated them to wear items to avoid mosquitoes. Participants also avoided going to the farm in late evening and/or nighttime. They will returned home before 6 o’clock in the evening.

Participants described the importance of having positive attitudes, including the motivation to use any preventive practices to avoid mosquito bites/malaria and being consistent with its usage.

*“We have to take care of ourselves […] but for others, it is up to them. If they wanted to avoid the mosquitoes, they could avoid malaria too […] we must take care of ourselves! [we need to try our best to avoid contact with these mosquitoes […] Moreover, I do not want to be admitted to the hospital. I do not want to get sick, and then […] if I am sick, I have to seek medication […] So, we have to take care of ourselves* [Female, 45 years old, Kg Membantu Laut]

Participants from Kg Membatu Laut told of an older woman, who was never diagnosed with malaria infection, despite screenings done by the healthcare clinic. She is currently estimated to be over 100 years old. As stories circulated in surrounding villages, participants from Kg Tagumamal Darat provided similar descriptions. She was described as healthy (without known comorbidity) and has consistently used bed nets during sleep. She was seen gardening, farming, and even walking to the beach and going fishing.

##### Subtheme 2: Fear

Participants perceived that the continuous presence of mosquitoes affected them emotionally. They feel ‘mad’, and ‘angry’ [Female, 28 years old, Kg Paradason] with their nuisance. Even though there were various vector control methods, they did not effectively help them to avoid these mosquitoes, especially from outdoor biting. Even more, motivation to use these item consistenly and coping with their usage were equally affected because, at times, they feel “lazy” and “fed up” with the continous presence of mosquitoes [Female, 26 years old, Kg Paradason]. Nevertheless, these emotions supported their intention to avoid malaria.

While preventing mosquito bites was perceived as necessary, the benefits of preventing malaria were mentioned as a way to avoid being admitted to the hospital. Participants understood the need of admission when a person was diagnosed with malaria. This caused fear and worry, especially among the working husband and wife. Once admitted, the duration of absence will negatively impact household income. The fear of food scarcity and lack of support to care for their family members during their absence can cause them to experience guilt. Thus, avoiding malaria (or any disease in general) was a way to happiness, better health and livelihood.

“*I am worried if a mosquito bites me, and if I get malaria […] Then, I had to be admitted to the hospital. I am worried (for myself and my family), I do not want to get admitted. So, I have to avoid them […] if I get admitted, money will go to waste, instead of buying food, to buy other* necessities”. [Female, 40 years old, Kg Paradason]

##### Subtheme 3: Having the opportunity to stay indoors during the dark hours reduces exposure to the mosquito bites

**Fig. 11:**
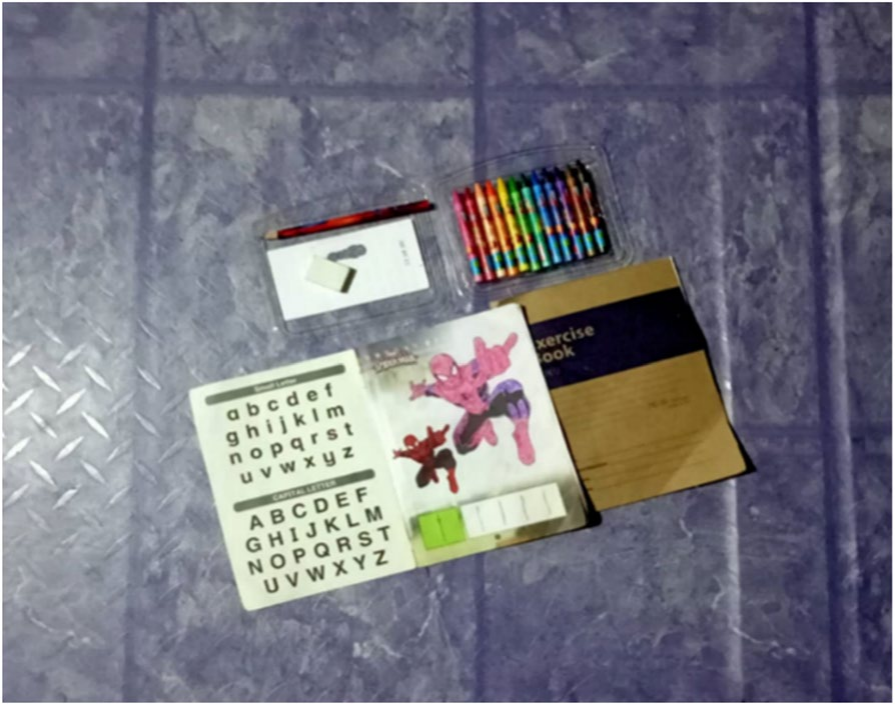
“*Around 8 until 8:30 o’clock at night, I usually teach my children to learn the alphabet and do coloring activities*” [Female, 29 years old, Kg Manduri]

Participants explained the lower exposure to malaria among women, was due to their daily housechores responsibilites, including taking care of their children and performing domestic work at night (e.g. preparing school clothes, cleaning the house, doing homework with children) (Fig. 11). By staying indoors, women reduced the risk of malaria by avoiding exposure to infective mosquito bites. The husbands mainly bore the livelihood and financial support. Participants highlighted that the neccesity of supporting their family and taking care of their children are significant roles as a housewife.

#### Theme 2: Social support

Participants emphasized the importance of social support in preventing malaria within their communities, which extended across households, neighborhoods and healthcare workers (HCWs). It was evident that the participants had familiar relationships with the HCWs such as being neighbors, relatives or spouses.

In households, women often have the opportunity to remain indoors, while their husbands typically go to work, even after nightfall. Within the neighborhood, the study areas consist of small communities, which facilitates the rapid spread of information. For instance, when malaria cases occur, the communities become more vigilant and regularly advise each other to stay alert.

Participants also highlighted the positive neighborhood practices aimed at continuously reminding one another about malaria prevention. Thee practices include sharingadvice, maintaining cleanliness in the surrounding neighborhood, and even repairing broken pipes for the gravity water system. They are aware of mosquitoes, and by assisting one another, they not only protect their own households but also helps fellow villagers avoid mosquitoes bite.

“*The community here always provides help in fixing the water pipes. They will gather and ensure no leakage in the pipes to allow a continuous supply of gravity water to the villagers’ houses. […] But, despite knowing the risk of mosquito bites and malaria […] they still need to walk and hike into the forest. That is where the water supply comes from, the gravity water*. [Female, 33 years old, Kg Membatu Laut].

#### Theme 3: Support from healthcare services and malaria awareness program

The support provided by HCWs and the primary care clinics was described as “sufficient.” Participants expressed general satisfaction with the services they received. Participants appreciated the ongoing awareness program, communication about health-related issues and updates on recent malaria cases. The distribution of bed nets, which were already treated with insecticide support their needs for protection against mosquitoes, especially during sleeping hours. The regular spraying of houses served as a constant reminder of the malaria risk in their villages. These efforts were seen as valuable to the communities, as they eliminated the need for communities to travel and purchase bednets themselves. However, participants mentioned that obtaining a new bed nets could be challenging in certain circumstances, even when the current one was torn and in need of replacement. They expressed hope that the stakeholder, in addition to the Ministry of Health (MOH) would support efforts to minimize the risk of “monkey malaria” in their villages.

## Discussion

This study demonstrates the effective use of photovoice in uncovering barriers and facilitators to malaria prevention in rural areas affected by *P. knowlesi* malaria in Sabah, Malaysia. The findings shed light on the diverse range of challenging barriers present in the physical and social environment, hinderingzoonotic malaria control efforts. However, individual factors such as the experiences of family members diagnosed with malaria and social support received through ongoing awareness programs conducted by health clinics emerged as facilitators in the fight against malaria.

This study reinforces the crucial role of collaboration with the community. The research team actively collaborated with and engaged the participants ensuring equitable power-sharing throughout the study. This approach fostered equity in research and democratization of knowledge [19]. When triangulated with different contexts (data, participants, methods), our findings could help identify suboptimal behaviors related to avoiding mosquito bites. Most importantly, the insights from the community at risk and individuals vulnerable to infection can inform the development of locally- tailored interventions [38, 39]. Maximizing the participation of communities in the design of *P. knowlesi* malaria intervention strategies may help accelerate incidence reduction. Recommendations include the provision of electricity and regular community consultations on control measures. The MOH should regularly update the community, academic partners, and stakeholders on disease updates, vector behavior and other pertinent epidemiological information.

It is clear that, for a large proportion of the rural communities, *P. knowlesi* malaria is perceived as a relatively minor concern when compared to other day to day challenges. Participants had a high degree of “acceptance” of mosquitoes' presence as a normal aspect of their lives. As a result, they become accustomed to coexisting with mosquitoes.

The study highlights the fact that despite the availability of various, tools, knowledge, and driving factors, there remain challenges to avoiding malaria in these communities. Factors such as local livelihood, lifestyle, and social environment contribute to community's risk of malaria exposure. The villages in this study have environments conducive to monkey malaria transmission. The *Anopheles balabacensis* mosquito larvae is a forest-dwelling species that prefers humid and shaded aquatic habitats [11]. The lack of electricity in the village provided a suitable “dark” environment for mosquitoes, increasing the risk of malaria exposure.

Within the context of social environment, there is a strong link between poverty and malaria [42]. This study revealed a link between low socioeconomic status and *P. knowlesi* malaria risk. Similarly, low socioeconomic status exposes rural communities in India [43], Madagascar, Mali and Nigeria to *P. falciparum* malaria, with a higher prevalence observed among those living in conditions of comparative poverty [26].

We have identified, despite having knowledge and awareness of the risks associated with malaria, many factors influence avoidance behaviour [25, 26] In some low and middle-income countries such as Indonesia, India, Thailand, Burkina Faso and Tanzania, traditional beliefs [44], limited accessibility to healthcare facilities providing preventive and diagnostic services [7, 45] ineffective health messages [46], low socioeconomic status [46], feasibility of the intervention [7, 43], affordability of intervention [43, 44], and religious, cultural and social gatherings [38, 47, 46] have all been linked with malaria exposure. Therefore, prioritization should be given to developing interventions that could help minimize malaria risk without ignoring the implications for livelihoods, social dynamics and cultural practices [48].

Policies and current malaria control measures need to reflect and incorporate the voices of community members. This study clearly shows that a conceptually sound malaria program may fail to be effective due to the unique local “microclimate” of the community. It is crucial to consider the broader consequences of interventions on communities. In order to make significant progress towards malaria elimination by 2030, there is a need to minimize the barriers while adopting new approaches to *P. knowlesi* malaria control. The design, implementation and evaluation of *P. knowlesi* malaria interventions should consider the challenges faced by the community as described in this study. Finally, a critical question for the future is “*How can we effectively minimize the social disparities in health for these vulnerable communities with respect to malaria?*”.

### Study limitations

Our study was based on the integration of participants' responses and our reflections throughout the research process. Therefore, it is important to note that the findings cannot be generalized to other geographical areas and/or populations. Although the sample size was small, the study's was strengthened by the rich and nuanced information that was shared by the participants. This photovoice study empowered participants [49] and revealed previously unseen areas for improvement and previously unexplored issues related to the communities’ lives, which relate to malaria exposure.” The ability to engage with policymakers and other stakeholders during the dissemination phases provided valuable opportunities to share the findings and invited participants to voice their perspectives. If traditional research methods had been employed, these oppurtunities would have been limited.

Our study was unable to establish causation or association between *P. knowlesi* malaria and community exposure. But it revealed in-depth insights into the barriers and facilitators of preventing mosquito bites. While the study is limited geographically, the identified barriers could potentially be extrapolated to similar rural communities where malaria control remains a problem. On the other hand, the facilitators could be constituted into future discussions, adopted or adapted in studies, and the development of new strategies for zoonotic malaria. The study used FGDs which limits the ability to conduct in-depth exploration with individual participants. A sequential one-to-one interview could be conducted to reveal more insights and stimulate more profound discussions with participants. Overall, the constrains of funding and time limited our ability to expand the study to other villages and/or involve a larger number of participants, which could have provided greater diversity in responses.

### Recommendations

Our study identified several areas for improving the public health strategy to control *P. knowlesi* malaria. First, there is a pressing need to improve the basic necessities and services in rural communities. Policymakers and stakeholders could support the development of treated water supplies, sewage systems, electricity and improvement of the communication infrastracture in these villages. Second, it is critical to support in improving the socioeconomic disparities as a means to alleviate the burden of malaria. Improving education levels can provide a solution to improve the work opportunities in the population, in view of the outdoor-work as a risk to malaria, faced by the rural population. In addition, supporting the development of social capacity through the integration of rural economy products into low and middle enterprises can have a positive impact. Third, the design of malaria interventions should consider the local social and cultural contexts to ensure their acceptability and effectiveness [46]. A greater focus on livelihood and rural community lifestyle could introduce a more locally suited preventive measure. A multi-collaborative efforts across sectors are critically needed in view of the broad spectrum of over-arching issues related to improving the health of population [18]. Participants expressed the belief that policymakers have a crucial role to play in providing additional resources to their community, including providing additional vector control items and improving their housing conditions. Given the limited focus of *P. knowlesi* malaria studies on the challenges faced by communities at risk, it is evident that greater attention, effort, and resources are required to reduce the burden among these communities.

## Conclusion

Social, cultural, environmental and structural factors are critical contributors of *P. knowlesi* malaria exposure. By conducting photovoice study with rural community members in Sabah, we gained valuable insights into their local perspectives and concerns. These findings can inform future malaria interventions, policies, and research endeavors. Our findings have broad implications for future research, development of comprehensive malaria strategies, and inform the necessity of improving local policies to establish a more feasible, accessible, and locally-relevant preventive methods for rural and vulnerable populations in Malaysia.

## Supplementary Information

**Additional file 1.** Summary description of participants.

**Additional file 2.** Interview questions.

**Additional file 3.** Thematic analysis with deductive-inductive approach.

## Data Availability

The datasets and photos generated from this study are available from the corresponding author on reasonable request.

## Abbreviations

P. knowlesi: *Plasmodium knowlesi*
WHO: World Health Organization
M: *Macaque*
ITNs: Insecticide treated bed nets
IRS: Insecticide residual spraying
MOH: Ministry of Health
CBPR: Community-based participatory research
Kg: Kampung
FGDs: Focus Group Discussions
FGD: Focus Group Discussion
