# Supplementary material for: Exploring barriers to and facilitators of malaria prevention practices: a photovoice study with rural communities at risk to Plasmodium knowlesi malaria in Sabah, Malaysia
Source: BMC Public Health. 2023 Jul 10;23:1316. doi: 10.1186/s12889-023-16173-x (PMC10332084; doi:10.1186/s12889-023-16173-x)
Supplement: Supplementary file 1 — Additional file 1. Summary description of participants. [file 12889_2023_16173_MOESM1_ESM.docx]

Additional File 1. Summary description of participants

| Participants | Identity | Age (years) during the study year in 2022 | Main occupation | History of malaria infection |
| --- | --- | --- | --- | --- |
| Kampung Manduri |  |  |  |  |
| 1 | KM001 | 55 | Farmer | No |
| 2 | KM002 | 40 | Farmer | Yes |
| 3 | KM003 | 33 | Housewife | No |
| 4 | KM004 | 21 | Unemployed | No |
| 5 | KM005 | 32 | Farmer | No |
| 6 | KM006 | 29 | Housewife | No |
| Kampung Paradason |  |  |  |  |
| 1 | KP001 | 40 | Farmer | No |
| 2 | KP002 | 41 | Farmer | Yes |
| 3 | KP003 | 22 | Housewife | Yes |
| 4 | KP004 | 24 | Housewife | No |
| 5 | KP005 | 28 | Farmer | Yes |
| 6 | KP006 | 27 | Housewife | No |
| Kampung Tagumamal Darat |  |  |  |  |
| 1 | KT001 | 46 | Farmer | Yes |
| 2 | KT002 | 44 | Farmer | No |
| 3 | KT003 | 62 | Farmer | Yes |
| 4 | KT004 | 33 | Gardener at school | No |
| 5 | KT005 | 27 | Farmer | No |
| 6 | KT006 | 35 | Housewife | No |
| 7 | KT007 | 53 | Farmer | No |
| 8 | KT008 | 72 | Farmer | Yes |
| Kampung Membatu Laut |  |  |  |  |
| 1 | KL001 | 33 | Farmer | No |
| 2 | KL002 | 35 | Housewife | No |
| 3 | KL003 | 33 | Unemployed | No |
| 4 | KL004 | 34 | Medical lab technologist | No |
| 5 | KL005 | 63 | Farmer | No |
| 6 | KL006 | 45 | Farmer | No |
| Total participants 26 persons | - | Median age = 34.5 | - | 7/26 = 26.92% |
