# Supplementary material for: Exploring barriers to and facilitators of malaria prevention practices: a photovoice study with rural communities at risk to Plasmodium knowlesi malaria in Sabah, Malaysia
Source: BMC Public Health. 2023 Jul 10;23:1316. doi: 10.1186/s12889-023-16173-x (PMC10332084; doi:10.1186/s12889-023-16173-x)
Supplement: Supplementary file 2 — Additional file 2. Interview questions. [file 12889_2023_16173_MOESM2_ESM.docx]

Additional File 2.

Research title: Exploring barriers to and facilitators of malaria prevention practices: A photovoice study with rural communities at risk to Plasmodium knowlesi malaria in Sabah, Malaysia.

Research board approval: Medical Research and Ethics Committee, Ministry of Health Malaysia (NMRR ID- 21- 01980- JEH), and the Research and Innovation Secretariat, Faculty of Medicine, Universiti Kebangsaan Malaysia (FF- 2021- 462).

Interview questions:

| Research objectives | Question draft | Piloted questions |
| --- | --- | --- |
| To explore and document the lived experiences and perspectives related to barriers and facilitators of preventing malaria among people living in rural villages exposed to *P. knowlesi* malaria in Kudat, Sabah, Malaysia | How a person could be infected with malaria? | Why could a person in your village get malaria?  Probing  1.At which area/location can a person be exposed to mosquito bites in your village?  2. When can a person be bitten by a mosquito that brought malaria?  3. Who is at risk of malaria in your village?  4. What activities exposed a person in your village to mosquito bites?  5. What activities do you perform from the late evening (around 6 o’clock) until morning (6 o’clock)? |
|  | How can you or anyone in your village could prevent mosquito bites? | How can you or anyone in your village prevent from getting mosquito bites?  Probing question  1. Why do you or anyone in your village prefer to use (the item e.g. mosquito repellents?)  2. Why do you or anyone in your village not like/prefer (e.g., sleeping under the bednet?)  3. Why is it difficult/challenging to avoid mosquito bites in your village?  4. Why a person or yourself could avoid mosquito bites?  5. What do you think of other ways to prevent mosquito bites/malaria in your village? |
