# Supplementary material for: Exploring barriers to and facilitators of malaria prevention practices: a photovoice study with rural communities at risk to Plasmodium knowlesi malaria in Sabah, Malaysia
Source: BMC Public Health. 2023 Jul 10;23:1316. doi: 10.1186/s12889-023-16173-x (PMC10332084; doi:10.1186/s12889-023-16173-x)
Supplement: Supplementary file 3 — Additional file 3. Thematic analysis with deductive-inductive approach. [file 12889_2023_16173_MOESM3_ESM.pdf]

Additional File 3: Thematic analysis with deductive-inductive approach.

Themes (deductive approach)	Themes derived from the Delphi study and its definition	FGD contents (with codes)
Barriers		
Intrapersonal factors	1. Demographic factor: Demographic factors are age, gender, occupation, education, and household income that possibly influence malaria preventive behavior.<br><br>2. Cognitive factor: Cognitive factors describe how the mental process acquires knowledge and understanding about something through thought, experience, reasoning and language. This includes the attitude, ability or inability to identify risky behaviors to malaria, perceive threat and vulnerability towards the malaria infection and subjective norms (perceive others' thinking), and. For example: How does a person/community believe a person can get the malaria infection or how does wearing long pants and shirts could protect a person from getting malaria?<br><br>3.Knowledge: Knowledge is a justified belief (e.g through awareness programme, reading, education, health promotion) that describes what the individual and community understand about the disease, transmission, activities at risk, and protective measures, including wearing protective clothing when performing outdoor work and putting on bednets during sleep.	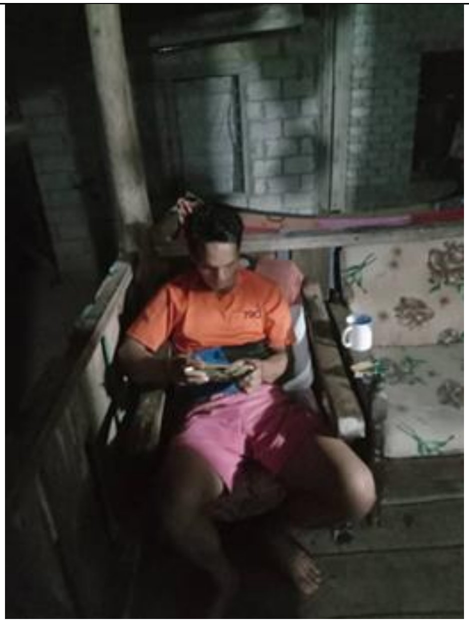<br>*"Relaxing at the verandah after dinner while using the smartphone." [Farmer, 32 years old. Kg. Manduri] (male gender, low perceive of risk, attitude, activity, lifestyle)*

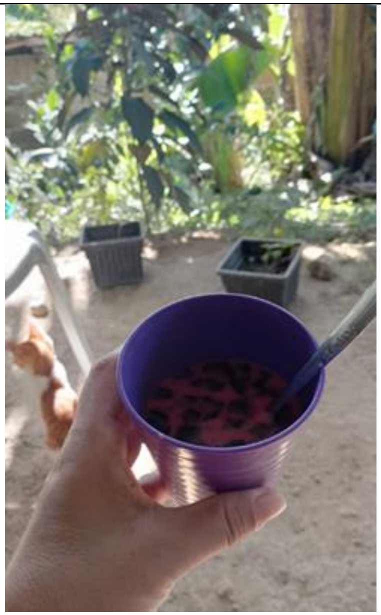

“My husband, he enjoys having a cup of coffee. In the evening, he relaxes outside the house while drinking Bandung syrup with grass jelly.” *[*Gardener, 33 years old, Kg. Tagumamal Darat] (activity, lifestyle, leisure)

"*My husband got malaria... He was wandering around there... He was hunting, in the neighboring village... Over there... Paradason village*." [Farmer, 33 years old, Kg. Membatu Laut] (male gender, activities, low perceive of risk, attitude)

“*If it's like my husband,he cannot sit still. At 5 in the morning, even before the chicken wakes up, he is already up. That's how people in the village are. Everyone is always at work, they always find something to do*.” [Farmer, 33 years old, Kg. Membatu Laut] (male gender, activities, lifestyle)

		"*If it comes to malaria cases among women here, they are fewer... Men often like to go to the forest, enter the forest*." [Female, 55 years old, Kg. Manduri] (gender, activities, lifestyle)<br><br>"*From what I know, many people get malaria, especially those who go to farms, go to the river, especially at night. This includes children around the age of eight or nine years old. Men also get affected. However, malaria among women are rare, because they don't roam around freely. But for children, that are exposed (to malaria). Children around nine to ten years old, they go fishing from morning till night*." [Female, 55 years old, Kg. Manduri] (gender, age group, children at risk, activities, lifestyle)<br><br>"*Rubber tapping is one of the activities that can contribute to the occurrence of malaria in individuals, as most of them engage in rubber tapping during the night, when it is suitable for latex production and when mosquitoes are also active*." [Housewife, 35 years old, Kg. Tagumamal Darat] (activities, environment, occupation, influence of time]<br><br>"*Men enjoy hanging out and relaxing*." [Housewife, 29 years old, Kg. Manduri] (male gender, activities, social norm)<br><br>Interviewer: "*What is the purpose of hunting in this village?.*"<br>Participant: "*It is for personal needs, and if there is excess, they can sell it for extra money. It is their essential livelihood... a combination of livelihood and hobby*."[Housewife, 35 years old, Kg. Tagumamal Darat] (gender, activities, lifestyle, livelihood)

		"*If we use mosquito repellent, the animals will run away because of the smell. If we inhale it, it's not good. They [the hunted animals] can smell it from afar. They will stay away from our location if they smell it... This hunting activity... If there's no work during the day, they go at night... It's boring, but they still go*." [Farmer, 45 years old, Kg. Membatu Laut] (activities, lifestyle, social norm)<br><br>"*They run away, those animals... They can smell... Those animals, even from a distance, they can smell it... When they don't have nothing else to do, when they feel boring, they will go hunting. That's how it is, always like that.*" [Farmer, 33 years old, Kg. membatu Laut] (activities, lifestyle, social norm, environment)
The environment	Environmental factors:<br>Environmental factors are extrinsic factors that may influence the malaria preventive behavior, for example:<br>1. Physical environment: This environment can be subdivided into uncontrolled/natural or<br>controlled/artificial. The former include the land condition, water, plants, forests, animals and the later include other infrastructure/buildings, for example the community house (e.g. house structure and<br>characteristic). Peridomestic condition can either be controlled/uncontrolled. It is the<br>surrounding area of the individual house, including long grass, paddy fields, water ponds, oil palms, presence of macaque monkeys,<br>mosquitoes and others). For example, "village-houses" are commonly built with rattan or<br>bamboo, present open eaves on the wall, and provide ways for the mosquito to fly indoors. Also, the presence of	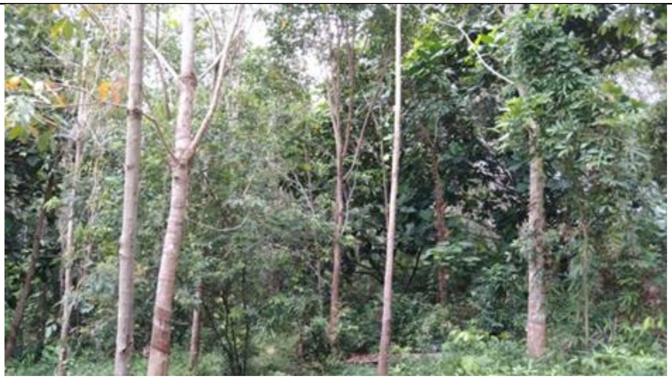<br>*"In the evening, around 6 o'clock, I go and to visit the rubber plantation to inspect each tapped rubber tree."[34 years old, medical lab technologist, Kg. Membatu Laut] (environment, livelihood, lifestyle)*

	mosquitoes, natural reservoir (e.g. Macaque monkeys), forests, plantation, farming land that serve as suitable condition for malaria transmission.<br>2. Social environment: As defined by Barret and Casper (2001) as "the immediate physical surroundings, social relationships, and cultural milieus within which defined groups of people function and interact" The components include built infrastructure; industrial and occupational structure; labor markets; social and economic processes; wealth; social, human,and health services; power relations; government; race relations; social inequality; cultural practices; the arts; religious institutions and practices; and beliefs about place and community; for example, human work/activities including timing or duration of work/activities that exposed them to infective mosquito bites, travelling duration to work/activities that poses similar exposure to malaria, frequency of going to the work/activities (e.g in the jungle) and details on agricultural or outdoor activities.<br>3. The influence of community value and social norms (nonverbal rules of behavior that are acceptable in a group or community). For example, how do the community members support each other by advising to avoid outdoor activities during nighttime, due to the risk of mosquito bites?<br>4.Socioeconomic factors that include details on their source of income, e.g location, timing, effect of weather, environment and others that could influence their malaria preventive behavior.	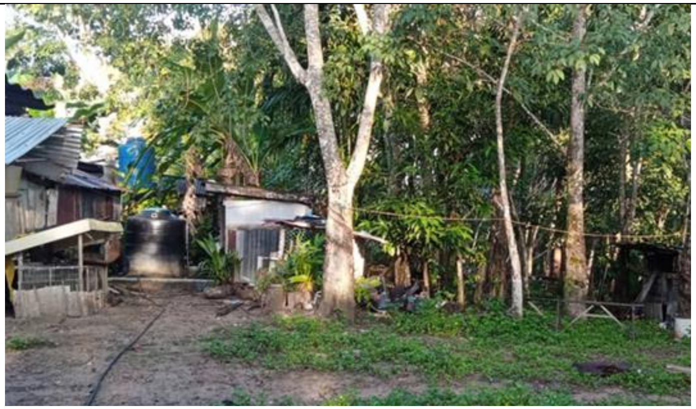<br>*"The area around the house is where my relative and myself go to, as a place to hang out." [Unemployed, 21years old, Kg Manduri] (activities, environment)*<br><br>"*There are many monkeys near the riverbank, especially coconut trees. Monkeys eat coconuts as their food*." [Farmer, 55 years old, Kg. Manduri] (monkey presence, environment)<br><br>"*There are many mosquitoes in the morning now (rainy season). During the dry season, there are usually no mosquitoes in the morning because the sun has risen. But if you're in the garden, there are always mosquitoes".* [Farmer, 40 years old, Kg. Paradason] (mosquito presence, environment, weather)<br><br>Participant: "*Mosquitoes are always present, so we are already accustomed to them... It is like... a norm of life*." [Housewife, 24 years old, Kg. Paradason] (a norm situation in the village, environment, mosquito presence)<br>Participant: "*It is not laziness... It is not that we do not want to use it (the mosquito control product), we try to avoid... but it is the norm of life... Having many mosquitoes is*

		normal... It is the norm of life here. We don't mind anymore." [Housewife, 22 years old, Kg. Paradason] (a norm situation in the village, environment, mosquito presence)<br>Participant: "*Yes, we don't mind" ".* [Farmer, 40 years old, Kg. Paradason] (mosquito presence, a norm situation in the village, environment)<br><br>Participant: "*It's not possible if we don't exercise... There's no specific place for jogging*." [Unemployed, 33 years old, Kg. Membatu Laut]<br>Participant: *"Activities like exercising, jogging... it is not possible if we do not do it, otherwise I will gain weight. Hahaha (laughing).]... So, we have to jog in the forest. Hahaha (laughing)."* [Housewife, 35 years old, Kg. Membatu Laut] (living a healthy life, determined, lifestyle, exercise)<br>Participant: "*And also, we must exercise, eventhough there is no proper place (indoor-exercise area), we can go exercise, even outside the house*." [Unemployed, 33 years old, Kg. Membatu Laut] (motivated, attitude, spirited, environment, health disparity, determined, living a healthy life, lifestyle)<br>Participant: "*There's no dedicated place for exercising in our village, it's inside buildings*." [Housewife, 35 years old, Kg. Membatu Laut] (Health and social disparity, feeling sad, despair, no proper area for exercise)<br><br>Participant: "*In this village, it's rare for people to wake up at 6 AM, they wake up even earlier*." [Farmer, 72 years old, Kg. Tagumamal Darat] (social norm)<br>Participant: "*I wake up at 5:30 o'clock in the morning*." [Gardener, 33 years old, Kg. Tagumamal Darat]<br>Participant: "*In the longhouse, around 5 o'clock in the morning, everyone is already heading to the farms. It is a daily activity and a norm here. If I sit at the end of the stairs*

		at my house, I can see people already going out. Around 4 in the morning. There are no people going out after 6 o'clock. None." [Farmer, 72 years old, Kg. Tagumamal Darat] (social norm, lifestyle)<br>Participant: "*Sometimes at 2 in the morning, there are people pounding rice."* [Farmer, 35 yers old, Kg. Tagumamal Darat
Drivers		
Theme 1:<br>Intrapersonal factors	1. Demographic factor: Demographic factors are age, gender, occupation, education, and household income that possibly influence malaria preventive behavior.<br><br>2. Cognitive factor: Cognitive factors describe how the mental process acquires knowledge and understanding about something through thought, experience, reasoning and language. This includes the attitude, ability, or inability to identify risky behaviors to malaria, perceive threat and vulnerability towards the malaria infection and subjective norms (perceive others' thinking), and. For example: How does a person/community believe a person can get the malaria infection or how does wearing long pants and shirts could protect a person from getting malaria?<br><br>3.Knowledge: Knowledge is a justified belief (e.g through awareness programme, reading, education, and health promotion) that describes what the individual and community understand about the disease, transmission, at-risk activities, and protective measures, including wearing protective clothing when performing outdoor work and putting on bednets during sleep.	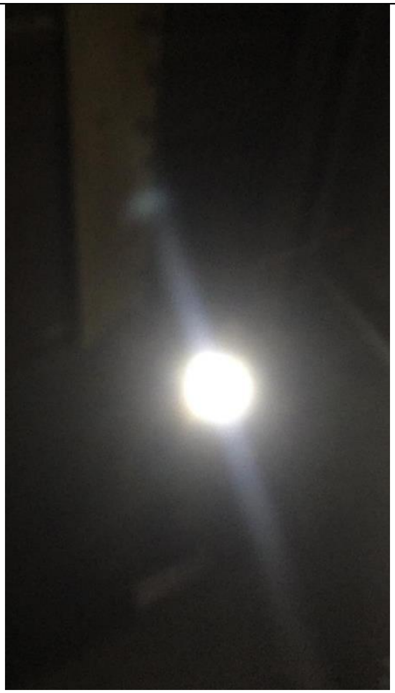<br>*"Always have the lights on at night to prevent mosquitoes from coming near us." [Farmer, 28 years old, Kg. Paradason] (perceive threat of malaria, knowledge)*

		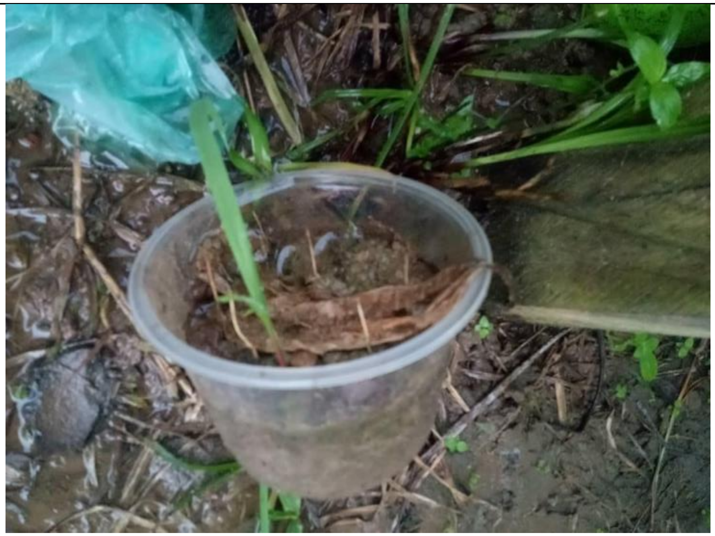<br>*"Clear the stagnant water to reduce the breeding of mosquitoes." [Farmer, 28 years old, Kg. Paradason] (perceive threat of malaria, knowledge, keeping the environment clean)*<br><br>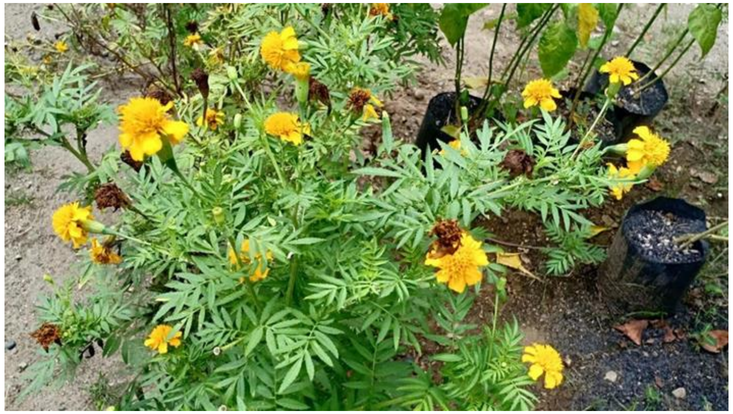<br>*"'Bunga tahi ayam' can repel mosquitoes due to their foul odor that mosquitoes dislike, and humans also find the smell unpleasant. Therefore, it is advisable to plant this flowers in the house yard." [Housewife, 24 years old, Kg. Paradason] (perceive threat of malaria, knowledge, a traditional way to prevent mosquito presence)*

		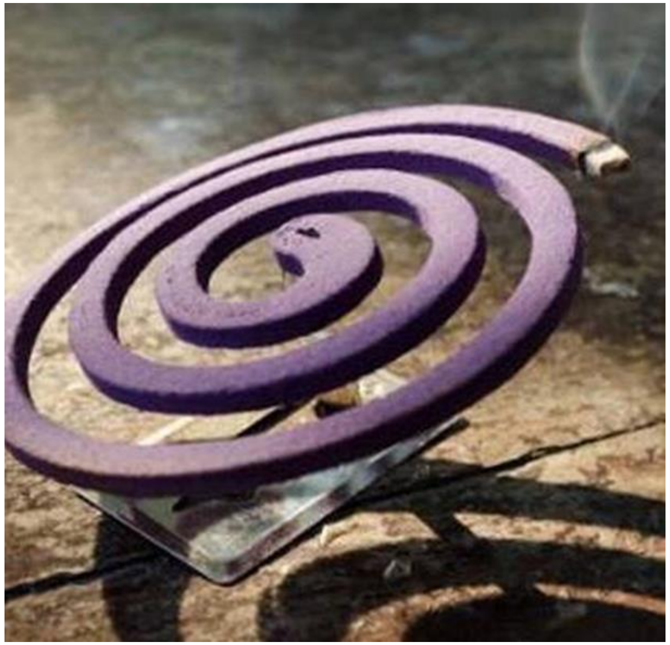 *"Always use mosquito repellent, both during the night when mosquitoes are active and during the day, to repel mosquitoes." [Housewife, 24 years old, Kg. Paradason] (perceive threat of malaria, knowledge, positive attitude)*<br><br>Participant: "*I cook inside the house... It is more comfortable to cook this way. I just chop it up (while demonstrating how she chopped the vegetables). My house has holes... therefore, to prevent mosquitoes... I use a mosquito coil and put it by my side (while cooking*)." [Farmer, 40 years old, Kg. Paradason] (perceive threat, avoiding nuisance of mosquitoes, positive attitude, feasible to be used while cooking, preferences of mosquito control products)<br>Participant: "*I always have that mosquito coil, cannott do without it (to avoid mosquito*)." [Housewife, 24 years old,

		Kg. Paradason] (preference to use the product to avoid mosquitoes, perceive threat, positive attitude)<br>Participant: "*Yes, usually when sleeping we use a mosquito net, but for cooking like this, the mosquito coil is suitable*."<br>)." [Farmer, 40 years old, Kg. Paradason] (perceive threat, avoiding nuisance of mosquitoes, positive attitude, feasible to be used while cooking, preferences of mosquito control products)
Theme 2: Social support	Social support describes the interpersonal interaction or support from the surrounding people, governance or other sectors, for example, by providing accessibility to mosquito-avoiding tools, or any effort or activities that helps to avoid mosquito.	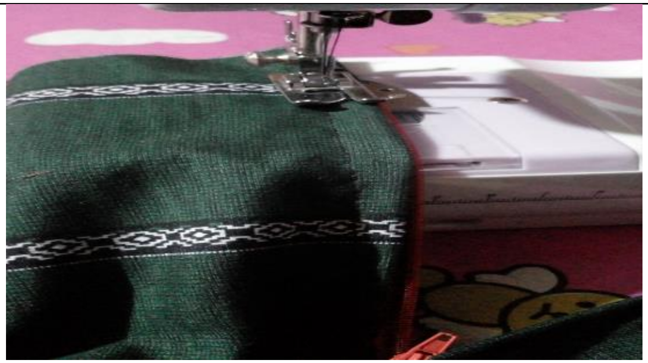<br>*"This is an evening activity where, after the children have gone to sleep, I take the opportunity to do some sewing. The picture shows me making a skirt." [Farmer, 44 years old, Kg. Tagumamal Darat]*

		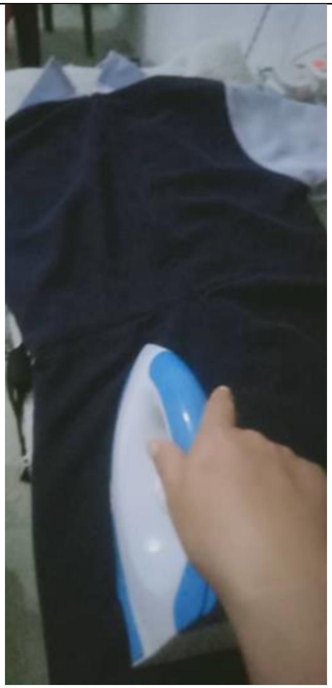<br>*"At 9 o'clock at night, I iron the school uniforms (of my children) in preparation for school tomorrow."[Gardener, 33 years old, Kg. Tagumamal Darat]*<br><br>"*Normally, the wives stay at home after sunset to take care of the house and children. The same goes for men if they do not have any specific reason to leave the house. However, if there is a family gathering, we gather in the open space of the house. Karaoke is a must. It directly strengthens family bonds... especially now, during the Kaamatan season, many people gather together."* [Housewife, 35 years old, Kg. Tagumamal Darat] (culture, social norm, gender preference, spouse support, activities with families, cultural tradition)

		Participant: "*In this village area, we do not have specific waste collection and disposal facilities. So, everyone just burns it (the rubbish).*" [Farmer, 46 years old, Kg. Tagumamal Darat] (social norms, finding alternative way to keep the village clean)<br>Participant: "*There is no proper disposal, it is difficult (to keep the area clean).*" [Farmer, 53 years old, Kg. Tagumamal Darat] (showing despair with the local governance or stakeholder support)<br>Participant: "*Usually, we have to find alternative methods. You just take it and burn it. It can be used to repel mosquitoes*." [Farmer, 46 years old, Kg. Tagumamal Darat] (social norms, finding alternative way to keep the village clean)<br><br>"*It is important to provide mosquito repellent or Ridsect assistance. The cost of purchasing it on their (the villagers) own is expensive, and the villagers cannot afford it. The standard of living in this village is such that they are unable to afford the necessary tools to prevent mosquito bites*." [Housewife, 29 years old, Kg. Manduri] (social support, inaffordability to purchase mosquito control products)

Themes (inductive approach)	Definition	FGD contents (with codes)
Barriers		
Theme 2: Livelihood and lifestyle activities	Livelihood and lifestyle activities refer to the various socioeconomic and daily life practices of the participants and rural villages in the study area that are influenced by and impact their ability to prevent malaria. This theme encompasses activities such as occupational practices, various income-generating activities, agricultural practices, social and cultural customs, and other aspects of their livelihoods and lifestyles that expose the individual and community to malaria and challenge malaria prevention	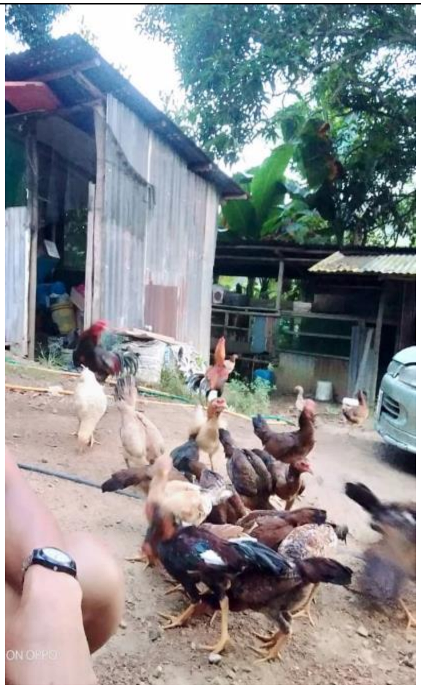<br>*"In the morning, around 6 o'clock, I feed my chickens." [Farmer, 53 years old, Kg. Tagumamal Darat] (activities, lifestyle)*<br><br>Participant: "*This is a picture of me feeding the chickens. It is one of my daily activities. I feed my chickens every morning at 6 am (while pointing to a photo he captured showing chickens)*" [Farmer, 53 years old. Kg. Tagumamal Darat]<br>Interviewer: *Why do you raise chickens?*

		Participants: "*I sell them to earn additional income*." [Farmer, 53 years old. Kg. Tagumamal Darat] (activities, lifestyle, livelihood)<br><br>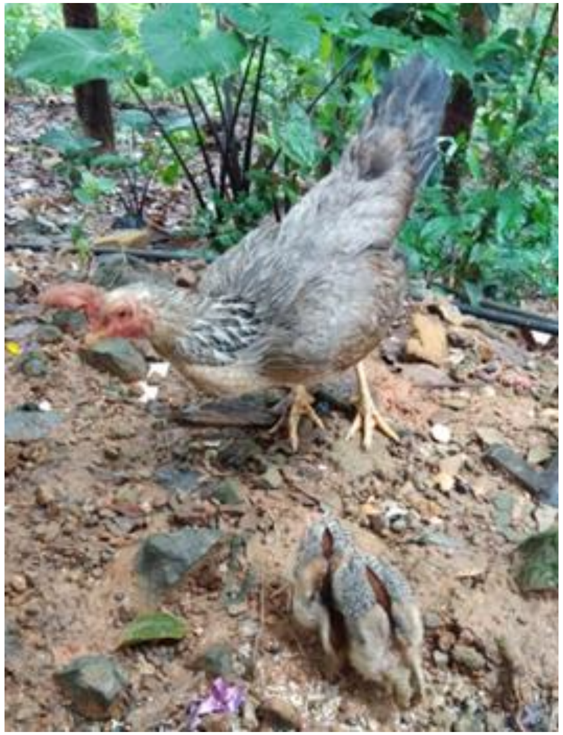<br>*"Feeding the chickens is a routine activity that is always done in the morning and evening."* [Farmer, 63 years old, Kg. Membatu Laut] *activities, lifestyle, social norm)*

		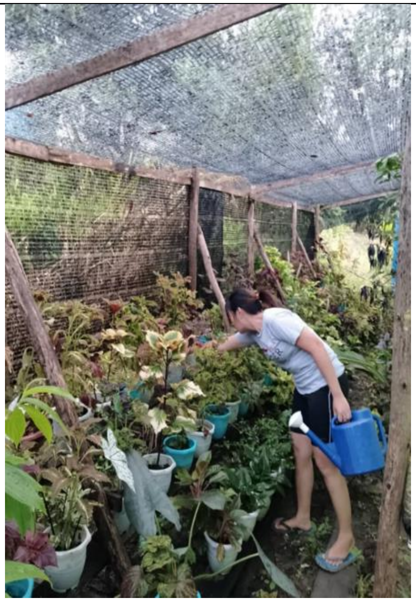 *"Every morning at around 6:30 o'clock, my way of spending leisure time is to visit the flower garden in my house yard to water the plants. At the same time, I will also remove any accumulated water under the pots and clean the wilted flower leaves. I will also pull out the weeds around the flower plants." [Housewife, 35 years old, Kg. Membatu Laut] (female gender, gardening, activities, lifestyle)*

		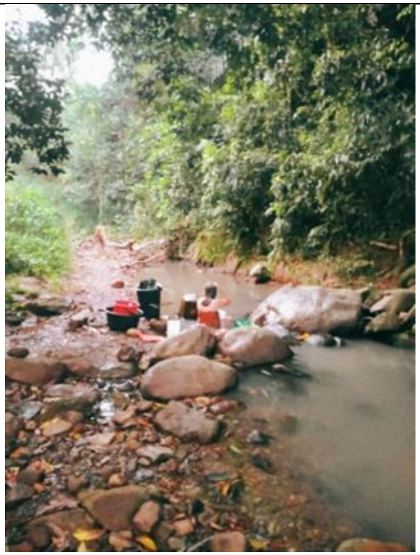<br>*“If there is no piped water available, they will take a bath in the river.” [Housewife, 35 years old, Kg. Membatu Laut] (social disparity, environment, limited access to treated water supply)*<br><br>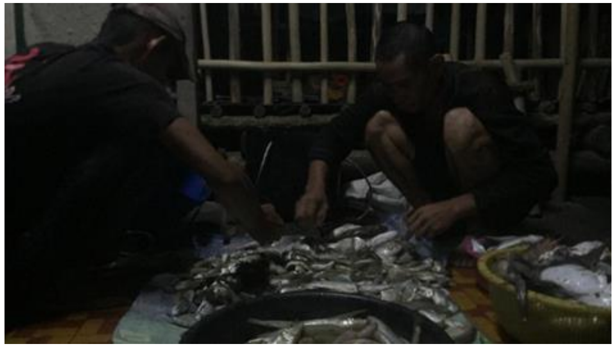<br>*“Activities from night time to 5 o’clock in the morning, include night fishing in the sea and tending the nets.” [Housewife, 24 years old, Kg. Paradason] (activities, livelihood, lifestyle, fishing activity)*

		Participant: "*He (husband) goes to tap rubber at night or in the morning depending on the time. Usually, after dinner, he starts walking through the forest, to the rubber farm. For example, at 7 o'clock at night, he takes a short rest and then walks to the rubber plantation at around 9 o'clock. He returns home after finishing.*" [Gardener, 33 years old, Kg. Tagumamal Darat] (occupation, livelihood, activity, environment)<br>Interviewer: "*Did he ever gets malaria?*"<br>Participant: "*Yes, during the Movement Control Order (MCO) last year, before the child, which had malaria earlier. He (husband) used to frequently drink coffee outside the house, especially during evenings and leisurely afternoons while sitting outside the house*." [Gardener, 33 years old, Kg. Tagumamal Darat] (leisure, hobby, lifestyle]<br><br>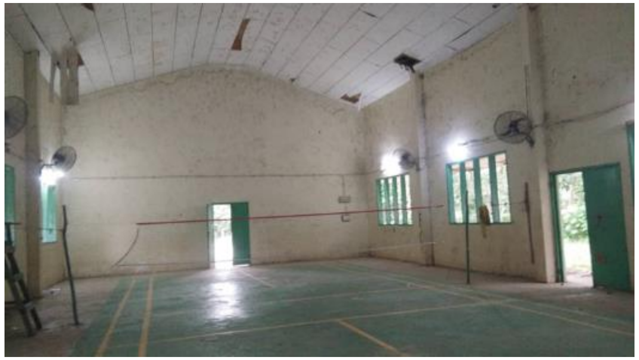<br>*"Playing sepak takraw or badminton is also one of the activities of the youth in the village during the evening until sunset." [Housewife, 29 years old, Kg. Manduri] (lifestyle, activities, social norm, youth)*<br><br>Participant (referring to the above photo showing the hall):"*They will play (badminton or sepak takraw) until*

		night." [Farmer, 40 years old, Kg. Manduri] (lifestyle, activities, social norm)<br>Participant: "*They stay (play at the hall) until 10 o'clock at night - they are active until then, both men and women, until 10 o'clock at night*." [Farmer, 55 years old, Kg. Manduri] (lifestyle, activities, social norm, both gender)
Drivers		
Theme 3: Support from healthcare services and malaria awareness program	Support from healthcare services and malaria awareness programs refers to the role of healthcare services, primarily the healthcare clinic and hospital in the study area. This theme also includes malaria-focused awareness programs in supporting individuals and communities in their efforts to prevent and control malaria, and empowering community members to prevent malaria . This theme explores the availability, accessibility, and quality of healthcare services and the effectiveness of malaria awareness programs in promoting preventive behaviors and raising awareness about malaria within the local population. In comparison to the 'social support' theme, this theme investigates how the healthcare providers engage with the local community, including the support and guidance in adopting preventive measures. It also assesses the impact of malaria awareness programs on individuals' knowledge, attitudes, and practices related to malaria prevention.	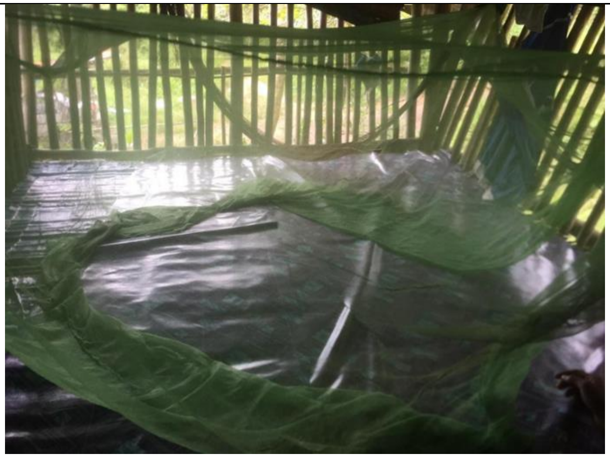<br>*"Putting on the mosquito net while sleeping at night to avoid mosquito bites." [Housewife, 27 years old, Kg. Paradason] (positive attitude, motivated, support from healthcare facilities/workers)*<br><br>"*If clothes (with insecticides) are washed, they cannot be worn again, but if it is sprayed, it can be sprayed multiple times. There was one before, I got a spray with a lemongrass scent (from the healthcare workers), but I could not use it. Its smell was too strong*." [Farmer, 40 years old, Kg. Manduri] (mosquito control product provided by the healthcare workers, promoted innovative ideas to avoid mosquitoes]

		”*When we sleep like this...we use mosquito nets (given by the healthcare workers) (pointing to the mosquito nets hanging on the wall, colored blue and green*).” [Housewife, 24 years ol,d, Kg Paradason] (awareness, perceive threat, bednets provided by healthcare workers)<br><br>“*Patients with malaria usually acquire the infection from areas with malaria cases such as forests and rubber plantations. The vector or carrier for malaria is the Anopheles mosquito. Anopheles mosquitoes will bite humans or monkeys infected with malaria parasites in their bloodstream and spread the infection by biting others. For example, in a rubber plantation, if an individual does not wear proper clothing or use mosquito repellent, they can be infected with malaria due to mosquito bites*.” [Medical lab technologist, 34 years old, Kg. Membatu Laut] [awareness of malaria, knowledge, perceive threat]
